# A novel method for generating glutamatergic SH-SY5Y neuron-like cells utilizing B-27 supplement

**DOI:** 10.3389/fphar.2022.943627

**Published:** 2022-10-20

**Authors:** Emily-Rose Martin, Josan Gandawijaya, Asami Oguro-Ando

**Affiliations:** ^1^ University of Exeter Medical School, University of Exeter, Exeter, United Kingdom; ^2^ Research Institute for Science and Technology, Tokyo University of Science, Tokyo, Japan

**Keywords:** SH-SY5Y cells, B-27 supplement, cell cycle, neuronal differentiation, glutamatergic, animal-component-free

## Abstract

The human SH-SY5Y neuroblastoma cell line is widely used in neuroscience research as a neuronal cell model. Following differentiation to a neuron-like state, SH-SY5Y cells become more morphologically similar to neurons and form functional synapses. Previous studies have managed to differentiate SH-SY5Y cells towards cholinergic, dopaminergic and adrenergic fates. However, their application in disease modeling remains limited as other neuronal subtypes (e.g., glutamatergic, GABAergic) are also implicated in neurological disorders, and no current protocols exist to generate these subtypes of differentiated SH-SY5Y cells. Our study aimed to evaluate the use of a xeno-free version of B-27, a supplement commonly used in neuronal culture, for SH-SY5Y maintenance and differentiation. To evaluate the proliferative capacity of SH-SY5Y cells cultured in B-27, we performed growth curve analyses, immunocytochemical staining for Ki-67 and qRT-PCR to track changes in cell cycle progression. SH-SY5Y cells cultured in FBS or under serum-starved conditions were used as controls. We observed that SH-SY5Y cells show reduced growth and proliferation rates accompanied by decreased *CDK6* and *CDK1* expression following 4-day exposure to B-27, suggesting B-27 induces a quiescent state in SH-SY5Y cells. Importantly, this reduced growth rate was not due to increased apoptosis. As cell cycle exit is associated with differentiation, we next sought to determine the fate of SH-SY5Y cells cultured in B-27. B-27-cultured SH-SY5Y cells show changes in cell morphology, adopting pyramidal shapes and extending neurites, and upregulation of neuronal differentiation markers (*GAP43*, *TUBB3*, and *SYP*). B-27-cultured SH-SY5Y cells also show increased expression of glutamatergic markers (*GLUL* and *GLS*). These findings suggest that B-27 may be a non-toxic inducer of glutamatergic SH-SY5Y differentiation. Our study demonstrates a novel way of using B-27 to obtain populations of glutamatergic SH-SY5Y cells. As dysregulated glutamatergic signaling is associated with a variety of neuropsychiatric and neurodegenerative disorders, the capability to generate glutamatergic neuron-like SH-SY5Y cells creates endless disease modeling opportunities. The ease of SH-SY5Y culture allows researchers to generate large-scale cultures for high-throughput pharmacological or toxicity studies. Also compatible with the growing popularity of animal-component-free studies, this xeno-free B-27/SH-SY5Y culture system will be a valuable tool to boost the translational potential of preliminary studies requiring glutamatergic neuronal cells of human origin.

## 1 Introduction

### 1.1 SH-SY5Y cell line as a neuronal model

An *in vitro* model widely used in neuroscience research is the human neuroblastoma SH-SY5Y cell line. SH-SY5Y is a thrice-subcloned cell line derived from the parental SK-N-SH neuroblastoma cell line, which was established in culture from metastatic cells found in the bone marrow of a four-year-old female neuroblastoma patient (Biedler et al., 1973; Xie et al., 2010; Kovalevich and Langford, 2013). SK-N-SH and SH-SY5Y cells exist as two morphologically distinct phenotypes: neuroblast-like or “N” type, and epithelial-like or “S” type (Ciccarone et al., 1989; Kovalevich and Langford, 2013). N-type SH-SY5Y cells possess many biochemical and functional properties of neurons (Kovalevich and Langford, 2013). For example, N-type SH-SY5Y cells express several neural markers and can synthesize various neurotransmitters (Ciccarone et al., 1989; Xie et al., 2010; Dwane et al., 2013). Furthermore, SH-SY5Y cells can be further differentiated in culture towards a more mature human neuronal phenotype (Påhlman et al., 1984; Encinas et al., 2000; Kovalevich and Langford, 2013; Teppola et al., 2016). In the differentiated form, SH-SY5Y cells appear more morphologically similar to human neurons, becoming more polarized, extending long neurites and forming functional synapses (Ciccarone et al., 1989; Encinas et al., 2000; Lopes et al., 2010). Moreover, differentiation of SH-SY5Y cells leads to their withdrawal from the cell cycle, producing a homogenous neuronal cell population (Påhlman et al., 1984; Encinas et al., 2000; Mollereau et al., 2007; Cernaianu et al., 2008; Guarnieri et al., 2009; Kovalevich and Langford, 2013).

Several methods to induce SH-SY5Y differentiation have been previously described, which involve a variety of differentiation-promoting factors (Påhlman et al., 1984; López-Carballo et al., 2002; Cernaianu et al., 2008; Kume et al., 2008; Cheung et al., 2009; Guarnieri et al., 2009; Kovalevich and Langford, 2013). One of the most implemented methods for SH-SY5Y cell differentiation involves treatment with Retinoic acid (RA) (Påhlman et al., 1984; López-Carballo et al., 2002; Cheung et al., 2009; Kovalevich and Langford, 2013; Riegerová et al., 2021). RA has been demonstrated to activate cell survival signaling pathways in SH-SY5Y cells, promoting cell survival and reducing susceptibility to neurotoxins (Encinas et al., 2000; López-Carballo et al., 2002; Cheung et al., 2009; Lopes et al., 2010). Several studies have also noted that the differentiation-promoting effects of RA can be further enhanced by treatment with Brain-derived neurotrophic factor (BDNF), resulting in elevated expression of neuronal marker genes and improved cell survival (Encinas et al., 2000; Cernaianu et al., 2008). Importantly, SH-SY5Y cells differentiate towards a primarily cholinergic neuronal phenotype following differentiation with RA, characterized by increased expression of Vesicular monoamine transporter 1 (VMAT1) and Choline acetyltransferase (CHAT) (Presgraves et al., 2003; Lopes et al., 2010). SH-SY5Y cells can also be differentiated towards other neuronal phenotypes, depending on the media conditions used (Kovalevich and Langford, 2013). For example, exposure to phorbol esters following RA treatment drives SH-SY5Y cells towards a dopaminergic neuronal phenotype, increasing expression of Tyrosine hydroxylase (TH) and Dopamine transporter (DAT) (Presgraves et al., 2003; Xie et al., 2010; Kovalevich and Langford, 2013). However, no studies to date have developed protocols to differentiate SH-SY5Y cells towards a glutamatergic phenotype.

### 1.2 Current advantages and limitations of SH-SY5Y cells

As a neuronal model cell line, SH-SY5Y cells have many advantages over other neuronal models. Not only can SH-SY5Y cells be differentiated towards a variety of adult neuronal phenotypes (Kovalevich and Langford, 2013), SH-SY5Y cells are an immortalized cell line, meaning that they continue proliferating (Xie et al., 2010; Kovalevich and Langford, 2013). Unlike primary neurons and induced pluripotent stem cell (iPSC)-derived neurons, SH-SY5Y cells retain the capacity for large-scale expansion, making them a suitable and cost-effective model (Kovalevich and Langford, 2013; Forster et al., 2016). Accordingly, SH-SY5Y cells are widely used in screening studies to assess the neurotrophic properties and neurotoxicity of pharmaceuticals (Datki et al., 2003; Henkel et al., 2008; Xie et al., 2010). Moreover, SH-SY5Y cells are simpler to genetically modify than animal models or primary neurons, so SH-SY5Y cells provide an excellent system for investigating the impact of disease candidate genes on neuronal processes (Bell and Zempel, 2021). Importantly, SH-SY5Y cells are of human origin, expressing many human-specific proteins and protein isoforms not inherently present in rodent primary cultures (Kovalevich and Langford, 2013). Furthermore, as SH-SY5Y cells are considered a cell line, the extensive ethical issues associated with primary rodent and human neuronal culture do not apply to them (Kovalevich and Langford, 2013).

Despite the numerous benefits of SH-SY5Y cells, the use of this cell line as a neuronal model involves some limitations. One of the main disadvantages of SH-SY5Y cells is that in the undifferentiated state, SH-SY5Y cells in culture exhibit unsynchronized cell cycles and do not express many of the key markers of mature neurons (Påhlman et al., 1984; Kovalevich and Langford, 2013). Moreover, undifferentiated SH-SY5Y cells have been shown to express several markers of immature neurons, and even markers of glial cells and their progenitors [e.g., Vimentin (VIM), Achaete-Scute family BHLH transcription factor 1 (ASCL1) and Stathmin 1 (STMN1)] (Messi et al., 2008; Byrne et al., 2013; Kovalevich and Langford, 2013; Ruangjaroon et al., 2017; Zammit et al., 2018). This suggests that in the undifferentiated state, SH-SY5Y cells may be more appropriate as a model of neural progenitor cells, and that their differentiation is required in order to recapitulate the molecular processes and functions of mature neurons. There are also limitations with differentiating SH-SY5Y cells. Previous studies have demonstrated that SH-SY5Y cells can be differentiated towards a cholinergic, dopaminergic or adrenergic phenotype (Kovalevich and Langford, 2013). However, there are currently no established protocols to differentiate SH-SY5Y cells towards other mature neuronal phenotypes, including glutamatergic and GABAergic neurons, and this considerably reduces their application in neuroscience research. Furthermore, despite being a human-derived cell line, the majority of SH-SY5Y fundamental culture methods use animal-derived components or supplements, such as fetal bovine serum (FBS) (Kovalevich and Langford, 2013; Xicoy et al., 2017). Therefore, the use of human SH-SY5Y cell lines to increase the human relevance or impact of neuroscience research becomes juxtaposed by the continued use of animal-derived biomaterials. To increase the versatility of the SH-SY5Y cell line in neuroscience research, it is necessary to look for alternative culture methods to generate other mature neuronal phenotypes, as opposed to the current methods using animal-derived biomaterials.

### 1.3 B-27 supplement

B-27 supplement is a serum-free neuronal cell supplement originally developed by Brewer et al., 1993 (Brewer and Cotman, 1989; Brewer et al., 1993). B-27 was designed to improve the survival of primary neurons in culture, and has since been used for maintenance, maturation and differentiation of neural stem cells and stem cell-derived neurons (Brewer and Cotman, 1989; Brewer et al., 1993; Chen et al., 2008). The classic B-27 supplement is defined as a mixture of vitamins, proteins, fatty acids and antioxidant enzymes that promote long-term viability of neurons (Brewer and Cotman, 1989; Brewer et al., 1993). Furthermore, several nutritive variants of B-27 have been developed to expand the applicability of B-27 in neuroscience research, including an antioxidant-free formulation to study neuronal oxidative stress, and an insulin-free formulation to study insulin secretion (B-27 Supplement: The Standard for Neuronal Cell Culture (Thermo Fisher Scientific UK, 2022)). The use of chemically defined supplements such as B-27 has several advantages over serum supplements. Using chemically defined growth culture supplements as opposed to serums, which can exhibit batch-to-batch variation, reduces the variability of cell culture conditions and eliminates the risk of undefined components unknowingly affecting the health of cell cultures (Chen et al., 2008). Additionally, a xeno-free formulation of B-27 (*Thermo Scientific*, #A1486701) has been developed which contains no animal-derived components at the primary component level, which may increase the translational potential of neuroscience research. This xeno-free B-27 is comprised of defined humanized and recombinant proteins, which further reduces batch-to-batch variation and data variability. In this study, we evaluate the effects of xeno-free B-27 on SH-SY5Y proliferation, differentiation and neurite outgrowth.

## 2 Materials and methods

### 2.1 SH-SY5Y cell culture

Human neuroblastoma SH-SY5Y cells (*American Type Culture Collection*, #CRL-2266™) were thawed from frozen stocks stored in 10% dimethyl sulfoxide (*Sigma Aldrich*, #D2650-5X5ML) at −80°C. SH-SY5Y cells were cultured in 10-cm diameter Petri dishes (*Sigma Aldrich*, #P7612-360EA) with 10 ml Dulbecco’s modified eagle medium/nutrient mixture F-12 with GlutaMAX (this is referred to simply as DMEM/F-12 in later sections) (*Fisher Scientific*, #11524436) supplemented with 10% FBS (*Fisher Scientific*, #11550356), and incubated at 37°C, 5% CO_2_, 95% humidity. Cells were passaged at 70% confluency using 3 ml of pre-warmed TrypLE™ (*Fisher* Scientific, #10718463) for 5 min and TrypLE™ was inactivated by dilution in an equal volume of DMEM/F-12 + 10% FBS. All cells used for experiments were of passage 15-18, and were passaged at least once after thawing before being used for experiments. For all experiments unless otherwise stated, SH-SY5Y cells were passaged and seeded into Nunc cell-culture treated 6-well plates (*Fisher Scientific*, #10469282) at a density of 300,000 cells per well in 3 ml DMEM/F-12 + 10% FBS. For most experiments, 6-well plates were not pre-coated with additional extracellular matrix factors. However, when pre-coating with additional extracellular matrix factors was performed, 6-well plates were pre-coated with 1 ml 20 μg/ml Poly-d-lysine (PDL) (*Fisher Scientific*, #11503550) overnight at 37°C, and washed thoroughly with ddH_2_0 prior to cell seeding.

In all experiments, DMEM/F-12 + GlutaMAX was used as the basal media. This was supplemented with either FBS, B-27, or small molecules and growth factors (e.g., RA and BDNF) which has been specified in the methods and figure legends.

### 2.2 Initial B-27 tests

**FIGURE 1:**
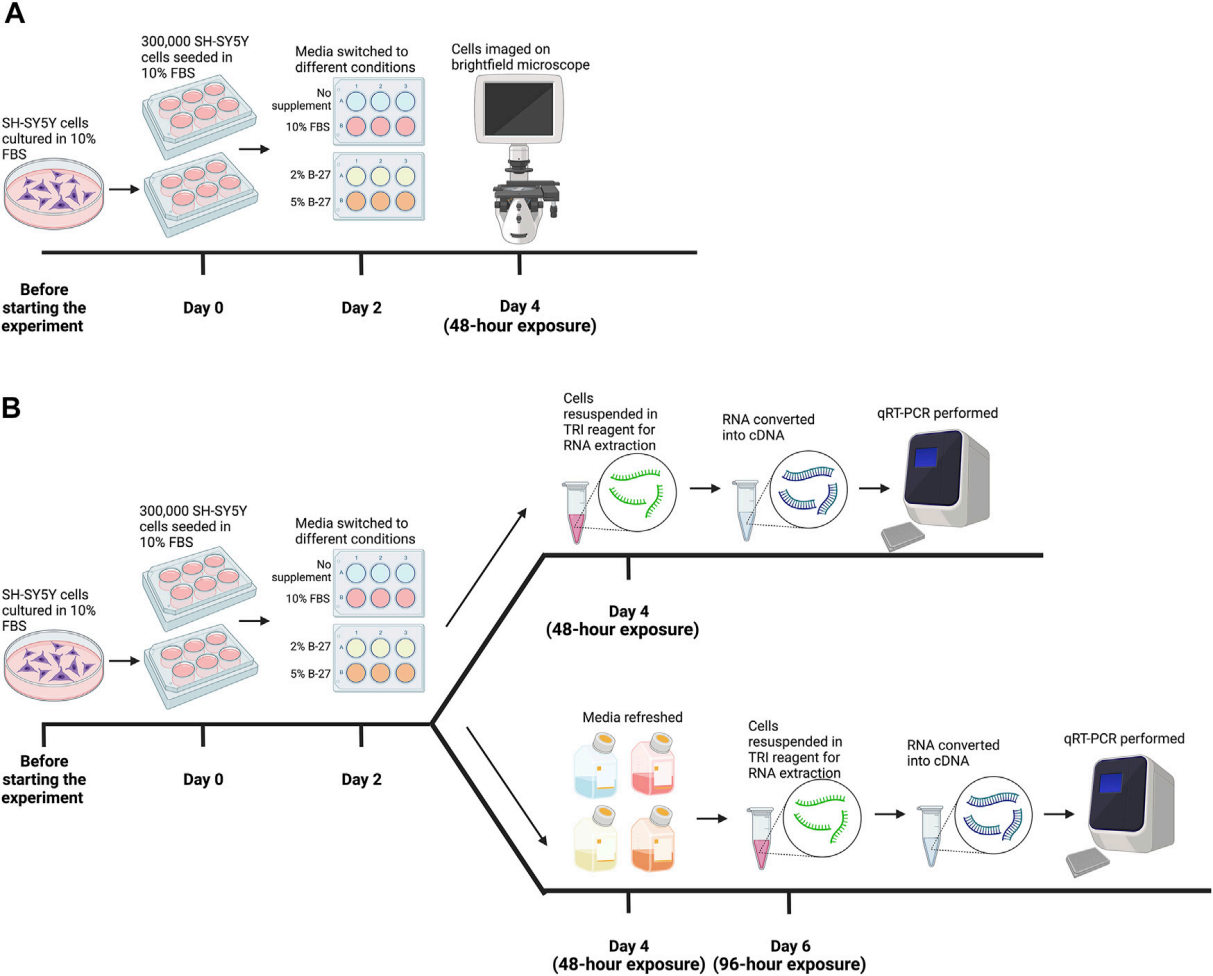
Methodology for testing B-27 for SH-SY5Y cell culture. **(A)** Initial B-27 tests. Before the start of the experiment, SH-SY5Y cells were cultured in DMEM/F-12 + 10% FBS in 10-cm diameter Petri dishes until they reached approximately 70% confluency. Once cells reached 70% confluency (day 0), 300,000 SH-SY5Y cells per well were seeded into 6-well plates in DMEM/F-12 + 10% FBS. By day 2, cells reached approximately 70% confluency and media was switched to either: DMEM/F-12 only, DMEM/F-12 + 10% FBS, DMEM/F-12 + 2% B-27 and DMEM/F-12 + 5% B-27. After 48 h (day 4), cells were imaged on the EVOS FLoid microscope (*Thermo Scientific*). **(B)** SH-SY5Y cells were cultured in the various media conditions for 48 h as described in part **(A)**. After 48 h (day 4), SH-SY5Y cells were either harvested in TRI reagent (*Zymo Research*) for RNA extraction, or the media was refreshed, and cells were cultured for a further 48 h. On day 6 (96-h exposure), SH-SY5Y cells were harvested in TRI reagent for RNA extraction. Following RNA extraction, RNA was converted into cDNA and qRT-PCR was performed on cells collected at both 48-h exposure and 96-h exposure time-points. DMEM/F-12, Dulbecco’s modified eagle medium/nutrient mixture F-12 with GlutaMAX supplement; FBS, fetal bovine serum; qRT-PCR, quantitative reverse transcription polymerase chain reaction. Figure made using BioRender.com.

SH-SY5Y cells were passaged and seeded into 6-well plates with or without PDL at a density of 300,000 cells per well, as described above. After 48 h, cells reached approximately 70% confluency and media was changed to either: DMEM/F-12 only, DMEM/F-12 + 10% FBS, DMEM/F-12 + 2% B-27 supplement or DMEM/F-12 + 5% B-27 supplement (see Figure 1A for further details). B-27 Supplement, XenoFree (*Fisher Scientific*, #13483269) was used in all experiments. Cells were imaged on the EVOS FLoid microscope (*Thermo Scientific*, #4471136) 48 h after the media was switched.

### 2.3 SH-SY5Y cell growth curve

On Day 0, SH-SY5Y cells were seeded into nine 6-well plates with 3 ml DMEM/F-12 + 10% FBS at a density of 20,000 cells per well. After 24 h (Day 1), media was replaced with 3 ml of either: DMEM/F-12 + 10% FBS, or DMEM/F-12 + 2% B-27 supplement. For each media condition, three wells were counted at the same time every day over 9 days. In order to perform cell counting, cells were dissociated with 500 μl TrypLE™ for 5 min and then an equal volume of the corresponding media was added. Ten microlitre of this cell suspension was then counted using a hemocytometer (*Fisher Scientific*, #10200872). For each well, four counts were performed, and an average was taken. Media was refreshed every 3 days.

### 2.4 Ki-67 staining and analysis

On Day 0, 5,000 SH-SY5Y cells were seeded into four 24-well plates in 500 μl DMEM/F-12 + 10% FBS. After 24 h (Day 1), media was replaced with 500 μl ml of either: DMEM/F-12 + 10% FBS, or DMEM/F-12 + 2% B-27 supplement. Media was refreshed every 3 days. Cells were fixed with 4% Paraformaldehyde (PFA) (Merck, #P6148-1kg) for 20 min at room temperature on Day 1, Day 3, Day 5 and Day 8. Cells were fixed in three independent wells per condition. The following steps were all preceded with three 10-min washes with 1× Phosphate-Buffered Saline (PBS) (Merck, #P4417-50TAB). Unless otherwise stated, all steps were performed at room temperature. After fixation, cells were permeabilized with 0.2% Triton X-100 (Merck, #T8787-100ML) in 1× PBS for 20 min, then blocked with 0.1% Triton X-100, 1% Bovine serum albumin (BSA) (Sigma-Aldrich, #A9647-50G) in 1× PBS for 1 h. Cells were incubated with 1:400 anti-Ki-67 (*Abcam*, #ab16667) overnight at 4°C, followed by incubation with 1:400 Alexa Fluor 555-conjugated donkey anti-rabbit (*Invitrogen*, #A31572). Nuclei were counter-stained with 1:5,000 4′,6-Diamidino-2-phenylindole (DAPI) (Merck, #D9542-1MG) for 5 min. Cells were imaged on the Leica DMi8 Widefield microscope (*Leica Microsystems*) at ×20 magnification.

The proportion of Ki-67 positive (Ki-67+) cells was analyzed using the *FIJI* software (Schindelin et al., 2012). The number of DAPI-stained nuclei and Ki-67+ cells per image were manually counted using the “Multi-Point” tool, to calculate the percentage of Ki-67+ cells per image. For each condition, three independent experimental replicates were performed, and 15 random locations in each well were analyzed.

### 2.5 RNA extraction, cDNA conversion and qRT-PCR

SH-SY5Y cells were cultured with either DMEM/F-12 only, DMEM/F-12 + 10% FBS, DMEM/F-12 + 2% B-27 supplement or DMEM/F-12 + 5% B-27 supplement for 48 h or 96 h (see Figure 1B for further details). Total RNA was isolated and purified from three independent wells per condition using the Direct-zol™ RNA Miniprep kit following manufacturer’s protocol (*Zymo Research*, #R2051). Five hundred nanograms of RNA was converted to cDNA using PrimeScript™ RT reagent kit (*Takara Bio Europe*, #RR037A) following manufacturer’s protocol. qRT-PCR was then performed using HOT FIREPol^®^ EvaGreen^®^ qPCR Master Mix with ROX (*Solis BioDyne*, #01-02-00500) with the QuantStudio 12K Flex qPCR machine (*Thermo Fisher Scientific*) on three biological replicates. For primer sequences, see Table 1. Messenger RNA (mRNA) expression levels were standardized against two reference genes, *Glyceraldehyde-3-phosphate dehydrogenase* (*GAPDH*) and *RNA polymerase II subunit A* (*POLR2A*) using the Pfaffl method (Pfaffl, 2001) and then normalized to the DMEM/F-12 + 10% FBS control samples. *GAPDH* and *POLR2A* were assessed as the most stable combination of genes (out of a panel of *GAPDH*, *POLR2A*, *PPIA* and *ATCB*) for use as endogenous controls in SH-SY5Y cells [performed using RefFinder, available at https://www.heartcure.com.au/reffinder/ (Xie et al., 2012)].

**TABLE 1:** qRT-PCR primers used in this study. All primers were reconstituted with PCR-grade ddH2O to 100 μM, before being used in qRT-PCR experiments at a concentration of 10 μM.

Gene	Primer type	Primer sequence (5′ → 3′)
*CASP3*	Forward	GGA​AGC​GAA​TCA​ATG​GAC​TCT​GG
	Reverse	GCA​TCG​ACA​TCT​GTA​CCA​GAC​C
*CDK1*	Forward	GGA​AAC​CAG​GAA​GCC​TAG​CAT​C
	Reverse	GGA​TGA​TTC​AGT​GCC​ATT​TTG​CC
*CDK6*	Forward	GGA​TAA​AGT​TCC​AGA​GCC​TGG​AG
	Reverse	GCG​ATG​CAC​TAC​TCG​GTG​TGA​A
*GAP43*	Forward	GAG​CAG​CCA​AGC​TGA​AGA​GAA​C
	Reverse	GCC​ATT​TCT​TAG​AGT​TCA​GGC​ATG
*GAPDH*	Forward	TCC​TCT​GAC​TTC​AAC​AGC​GAC
	Reverse	GCT​GTA​GCC​AAA​TTC​GTT​GTC​A
*GLS*	Forward	CAG​AAG​GCA​CAG​ACA​TGG​TTG​G
	Reverse	GGC​AGA​AAC​CAC​CAT​TAG​CCA​G
*GLUL*	Forward	CTG​CCA​TAC​CAA​CTT​CAG​CAC​C
	Reverse	ATA​GGC​ACG​GAT​GTG​GTA​CTG​G
*POLR2A*	Forward	CCA​TCA​AGA​GAG​TCC​AGT​TCG
	Reverse	ACC​CTC​CGT​CAC​AGA​CAT​TC
*SLC17A7*	Forward	GCA​AGT​ACA​TCG​AGG​ACG​CCA​T
	Reverse	GCC​ACG​ATG​ATG​GCA​TAG​ACT​G
*SLC18A1*	Forward	CAG​CCT​TCC​AAA​GTC​TCT​CCT​G
	Reverse	GCA​CAT​GGT​CTG​CAT​CAT​CCA​G
*SYP*	Forward	TCG​GCT​TTG​TGA​AGG​TGC​TGC​A
	Reverse	TCA​CTC​TCG​GTC​TTG​TTG​GCA​C
*TH*	Forward	GCT​GGA​CAA​GTG​TCA​TCA​CCT​G
	Reverse	CCT​GTA​CTG​GAA​GGC​GAT​CTC​A
*TUBB3*	Forward	TCA​GCG​TCT​ACT​ACA​ACG​AGG​C
	Reverse	GCC​TGA​AGA​GAT​GTC​CAA​AGG​C

### 2.6 Western blotting

SH-SY5Y cells were cultured in 10-cm diameter Petri dishes with DMEM/F-12 + 10% FBS until they reached approximately 60% confluency. Media was then switched to either DMEM/F-12 + 10% FBS, or DMEM/F-12 + 5% B-27 supplement and refreshed after 48 h. Ninety six hours after the initial media switch, cells were washed with ice-cold 1× PBS then collected with a cell scraper in 300 μl ice-cold RIPA lysis buffer (150 mM NaCl, 50 mM Tris/Cl pH = 8.0, 0.5% Sodium Deoxycholate, 0.1% SDS, 1% Triton X-100) supplemented with 1 mM PMSF, 1% Phosphatase Inhibitor Cocktail 2 (*Sigma Aldrich*, #P5726-1ML), 1% Phosphatase Inhibitor Cocktail 3 (*Sigma Aldrich*, #P0044-1ML) and 1% Complete Protease Inhibitor Cocktail (*Sigma Aldrich*, #P8340-1ML). Cells were collected from three independent Petri-dishes per condition. The cell lysates were incubated on ice for 30 min, then cleared by centrifugation at 13,200 rpm for 15 min in a precooled centrifuge at 4°C. The supernatant was collected, and Bicinchoninic Acid assays (*Thermo Scientific*, #10678484) were performed to estimate protein concentrations. Fifty micrograms of total protein was diluted with Laemmli buffer (final concentration of 60 mM Tris/Cl pH = 6.8, 10% Glycerol, 2% SDS, 5% 2-Mercaptoethanol, 0.02% Bromophenol Blue) and boiled for 5 min at 95°C. Proteins were separated in 10% SDS-PAGE gels and transferred onto PVDF membranes (*Merck*, #10796851). The following steps were all preceded with three 10-min washes with 1× Tris-Buffered Saline (TBS) + 0.1% Tween-20 (1× TBS-T). Membranes were incubated in blocking buffer (5% Skim Milk in 1× TBS-T) for 1 h at room temperature. Membranes were incubated with primary antibodies diluted 1:1,000 in blocking buffer overnight at 4°C or at room temperature for 1 h. Primary antibodies used: rabbit anti-GLS (*Proteintech*, #29519-1-AP), mouse anti-TH (*Proteintech*, #66334-1-Ig), mouse anti-GLUL (*Proteintech*, #66323-1-Ig) and mouse anti-GAPDH (*Santa Cruz Biotechnology*, #sc-47724). Membranes were incubated with corresponding secondary antibodies diluted 1:1,000 in blocking buffer at room temperature for 1 h. Secondary antibodies used: DyLight 800-conjugated goat anti-rabbit (*Thermo Scientific*, #SA5-10036) and DyLight 680-conjugated goat anti-mouse (*Thermo Scientific*, #A-2P57). Protein expression was visualized using the LI-COR^®^ Odyssey CLx imaging system. Densitometric analysis was performed using the *FIJI* software (Schindelin et al., 2012) to quantify levels of protein expression (relative to GAPDH expression).

### 2.7 Neurite outgrowth analysis

20,000 SH-SY5Y cells were seeded onto 13-mm diameter glass coverslips (*Fisher Scientific*, #11588492) pre-coated with 300 μl 20 μg/ml PDL overnight at 37°C in 24-well plates. Twenty four hours after seeding, media was aspirated and replaced with 500 μl DMEM/F-12 supplemented with either FBS, B-27 or small molecules and growth factors previously demonstrated to induce SH-SY5Y cell differentiation (Kovalevich and Langford, 2013), which has been specified in the respective figure legends. Media was refreshed every 48 h. Five days after seeding, cells were fixed with 4% PFA, permeabilized and blocked as described in Section 2.4. Coverslips were incubated with 1:500 mouse anti-β(III)-tubulin (TUBB3) (*R&D* Systems, #MAB1195) overnight at 4°C, followed by incubation with 1:400 Alexa Fluor 488-conjugated donkey anti-mouse (*Thermo Scientific*, #A21202) for 1 h. Nuclei were counter-stained with 1:5,000 DAPI for 5 min. Coverslips were mounted onto glass slides using DAKO mounting media (*Agilent Technologies*, #S302380-2). Slides were imaged on the Leica DM4000 LED upright fluorescence microscope (*Leica Microsystems*) at ×20 magnification.

Neurite tracing analysis was performed using the *FIJI* software (Schindelin et al., 2012). Prior to analysis, a scale of 320 pixels to 100 μm was applied to each image using the *FIJI* software. To determine neurite lengths, the “Segmented Line” tool was used to measure the distance from the border of the nucleus to the distal end of neurites. The sum of the lengths of all the neurites for each cell was used to provide a total neurite length measurement for each cell. For each condition, three independent experimental replicates were performed, and 80 cells were traced at random across the replicates.

### 2.8 Statistical analysis

For all experiments, data were analyzed, and statistical significance determined using GraphPad Prism version 9.0.0 for Mac, GraphPad Software, San Diego, California United States, www.graphpad.com. The differences between two groups were assessed by Unpaired *t*-tests. The differences between multiple groups were assessed by ordinary one-way ANOVA and Tukey’s multiple comparison tests. Data expressed as mean ± SEM. *p*-values < 0.05 were considered statistically significant.

## 3 Results

### 3.1 B-27 supplement encourages SH-SY5Y cell proliferation

**FIGURE 2:**
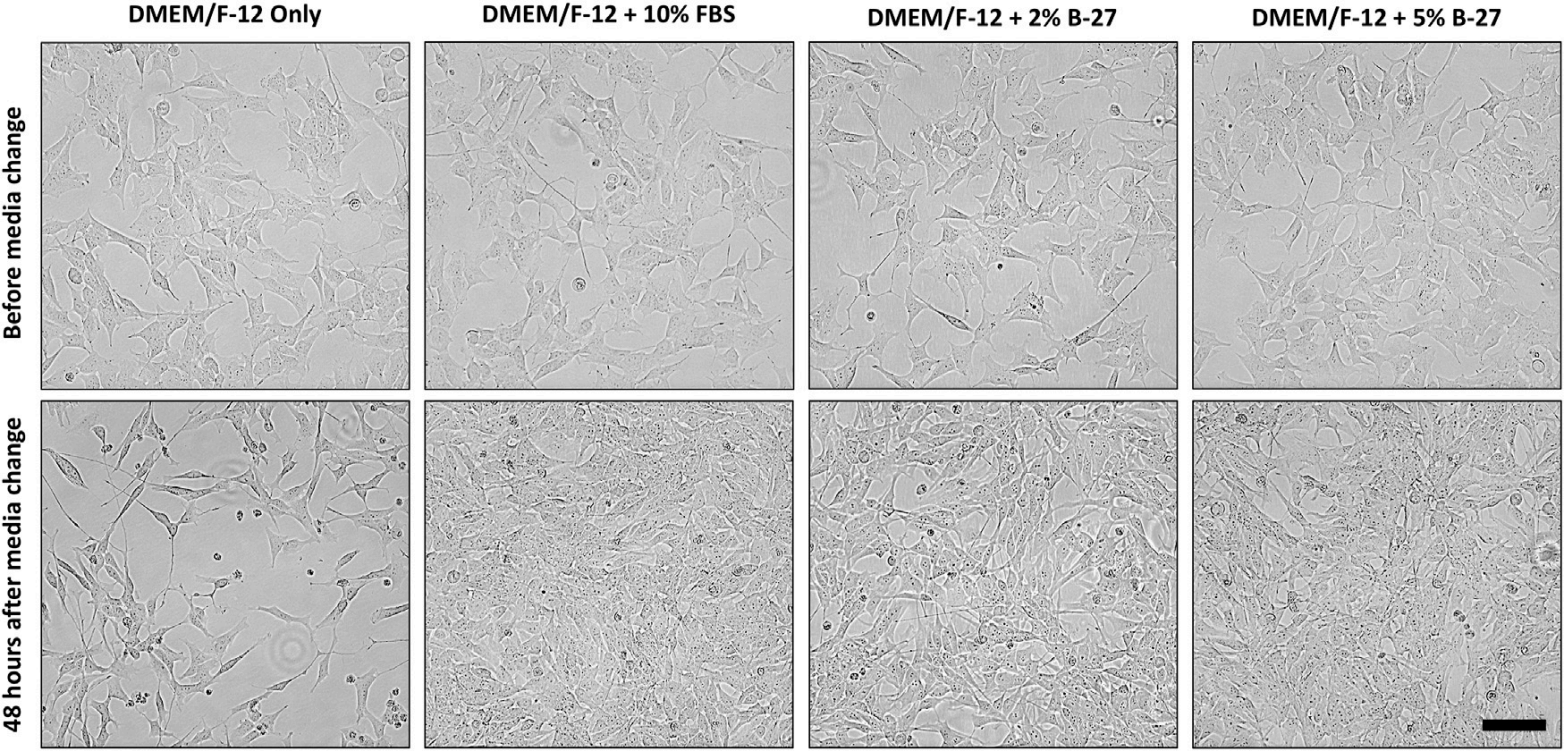
B-27 encourages SH-SY5Y cells were seeded into 6-well plates at a density of 300,000 cells per well in DMEM/F-12 + 10% FBS. Once SH-SY5Y cells reached approximately 70% confluency, media was replaced with either: DMEM/F-12 only, DMEM/F-12 + 10% FBS, DMEM/F-12 + 2% B-27 supplement or DMEM/F-12 + 5% B-27 supplement. Cells were imaged using the EVOS FLoid microscope (*Thermo Scientific*) before media was replaced and 48 h after media was replaced. Scale bar = 100 μm. DMEM/F-12, Dulbecco’s modified eagle medium/nutrient mixture F-12 with GlutaMAX supplement; FBS, fetal bovine serum.

To test whether B-27 could be a viable and effective substitute for FBS in SH-SY5Y cell culture, SH-SY5Y cells were cultured in DMEM/F-12 + 10% FBS until they reached approximately 70% confluency, and then the media was replaced with either DMEM/F-12 only, DMEM/F-12 + 2% B-27 or DMEM/F-12 + 5% B-27 for 48 h (Figure 2). We tested two different concentrations of B-27, 2%—which is the standard composition used for neuronal cell culture, and 5%—in the event that SH-SY5Y cells may need greater amounts of growth factors present in B-27 for subculture. As a negative control, DMEM/F-12 only was used, which demonstrated that serum starvation inhibits SH-SY5Y cell proliferation. We observed that SH-SY5Y cells cultured in B-27 were able to proliferate similarly to SH-SY5Y cells cultured in FBS. There was no observable difference in cell proliferation between the 2% B-27 and 5% B-27 conditions. This suggests that B-27 supplement promotes SH-SY5Y survival and proliferation.

### 3.2 SH-SY5Y cells show reduced proliferation following longer-term culture in B-27

**FIGURE 3:**
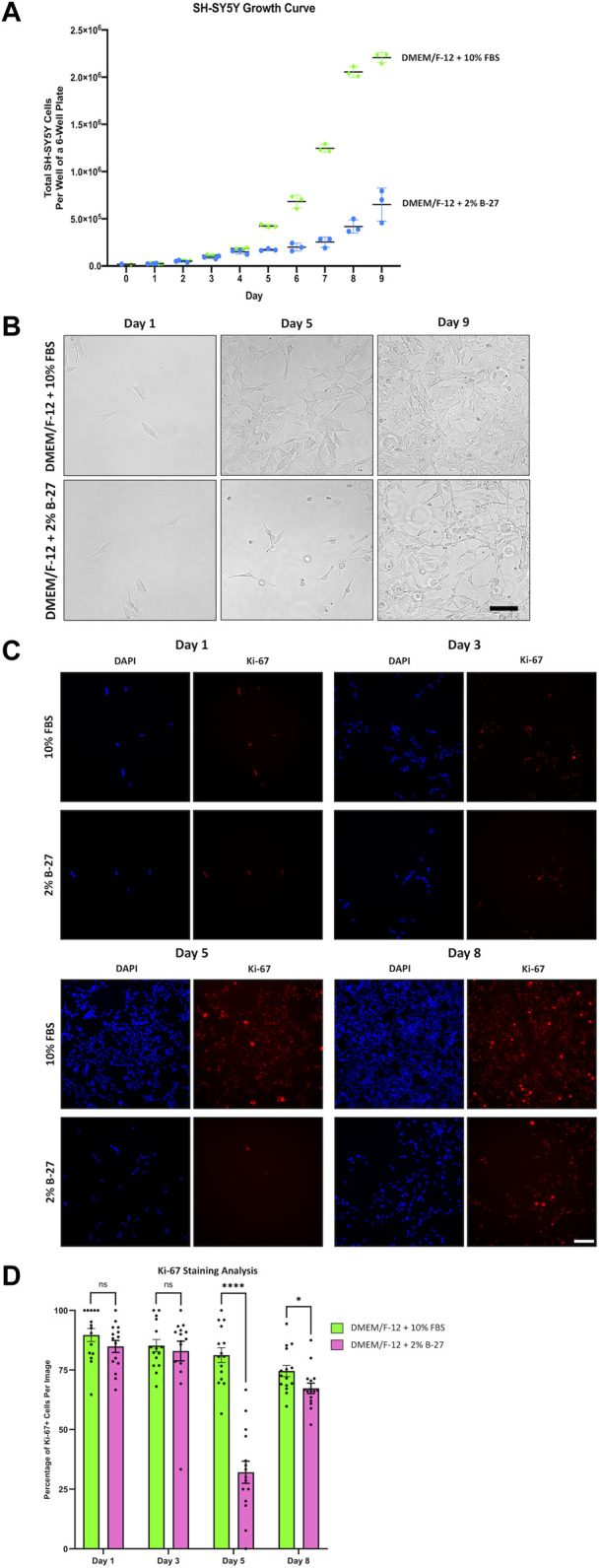
SH-SY5Y cells cultured in B-27 show reduced growth rate compared to SH-SY5Y cells cultured in FBS. **(A)** On Day 0, 20,000 SH-SY5Y cells per well were seeded into 6-well plates with DMEM/F-12 + 10% FBS. On Day 1, media was replaced with either DMEM/F-12 + 10% FBS or DMEM/F-12 + 2% B-27 supplement, and the number of cells per well was counted using a hemocytometer. Cell counts were performed at the same time daily for 9 days. **(B)** Representative brightfield mages were taken on Day 1, Day 5, and Day 9 using the EVOS FLoid microscope (*Thermo Scientific*). Scale bar = 100 μm. **(C)** On Day 0, 5,000 SH-SY5Y cells per well were seeded into 24-well plates with DMEM/F-12 + 10% FBS. On Day 1, media was replaced with either DMEM/F-12 + 10% FBS or DMEM/F-12 + 2% B-27 supplement. Cells were fixed on Day 1, Day 3, Day 5 and Day 8 with 4% PFA for 20 min. Proliferating cells were stained with 1:400 rabbit anti-Ki67 and visualized with 1:400 Alexa Fluor 555-conjugated donkey anti-rabbit (*Invitrogen*, #A31572) (red). Nuclei were counterstained with 1:5,000 DAPI (blue). Images taken at ×20 magnification through the DAPI (excitation 325–375 nm; emission 435–485 nm) and the TXR (excitation 540–580 nm; emission 592–668 nm) filter cubes on the Leica DMi8 Widefield microscope. Scale bar = 100 μm. **(D)** The number of Ki67+ cells and DAPI-stained nuclei were counted using the *FIJI* software, and the percentage of Ki-67+ cells per image calculated. Three independent replicates were performed, and 15 random images were analyzed per condition. Data presented as mean ± SEM. Statistical significance against the DMEM/F-12 + 10% FBS condition was determined using Unpaired *t* tests in GraphPad Prism version 9.0.0 for Mac, GraphPad Software, San Diego, California, United States, www.graphpad.com. ns = not significant; *p < 0.05; ****p < 1 × 10^–4^. DAPI: 4′,6-Diamidino-2-phenylindole; DMEM/F-12, Dulbecco’s modified eagle medium/nutrient mixture F-12 with GlutaMAX supplement; FBS, Fetal bovine serum; PFA, Paraformaldehyde; SEM, standard error of the mean.

Having demonstrated that SH-SY5Y cells were able to survive and proliferate in B-27 supplement, we wanted to compare the growth rates between SH-SY5Y cells cultured in DMEM/F-12 + 10% FBS or DMEM/F-12 + 2% B-27, so we generated growth curves for SH-SY5Y cultured in the different media conditions. 2% B-27 was chosen as there was no noticeable differences in proliferation between SH-SY5Y cells cultured in 2% B-27 and 5% B-27. We found that initially, SH-SY5Y cells cultured in 10% FBS and 2% B-27 showed no difference in growth rate (Figures 3A,B). However, after 5 days, when SH-SY5Y cells cultured in 10% FBS had clearly shifted to an exponential growth phase, SH-SY5Y cells cultured in 2% B-27 show a sudden reduction in growth rate. This suggests that whilst B-27 initially promotes SH-SY5Y cell proliferation, long-term exposure to B-27 (after 4 days) results in reduced proliferation.

In order to confirm these findings, SH-SY5Y cells were cultured in DMEM/F-12 supplemented with either 10% FBS or 2% B-27 for up to 8 days and were stained for Ki-67. Ki-67 is a widely used marker of cell proliferation (Sun and Kaufman, 2018). The nuclear Ki-67 antigen is only expressed in cells undergoing the active stages of the cell cycle (G1, S, G2 and M phases), and is absent in resting or quiescent cells (Sun and Kaufman, 2018). We observed that after 3 days, SH-SY5Y cells cultured in 2% B-27 showed no difference in the percentage of Ki-67+ cells compared to SH-SY5Y cells cultured in 10% FBS (*p* > 0.05) (Figures 3C,D). However, by day 5, B-27-cultured SH-SY5Y cells showed a significant reduction in the percentage of Ki-67+ cells compared to FBS-cultured SH-SY5Y cells (*p* < 1 × 10^–4^) (Figures 3C,D). This suggests that after 5 days exposure to B-27, SH-SY5Y cells enter a state of quiescence, with fewer cells actively progressing through the cell cycle. By day 8, SH-SY5Y cells cultured in B-27 continue to display a lower percentage of Ki-67+ cells compared to those cultured in FBS (*p* < 0.05), however the difference in percentage is much smaller compared to day 5 (Figures 3C,D). These findings indicate that although SH-SY5Y cells cultured in B-27 at first show similar numbers of actively proliferating cells compared to SH-SY5Y cells cultured in FBS, after 5 days B-27-cultured SH-SY5Y cells exhibit significantly lower numbers of actively proliferating cells, suggesting cell cycle arrest. Despite this, following 8 days culture in B-27, the proportion of Ki-67+ SH-SY5Y cells shifts again, increasing to become more similar to FBS-cultured SH-SY5Y cells. This mirrors the growth curve data, where cell number appears to begin doubling again after Day 7–8 onwards. We are unsure if this is due to a sudden re-entry into the cell cycle, suggesting B-27 only induces temporary cell cycle arrest, or if this is due to heterogeneity arising from S- and N-type SH-SY5Y cells responding differently to B-27.

### 3.3 Longer-term B-27 exposure leads to SH-SY5Y cell cycle arrest

**FIGURE 4:**
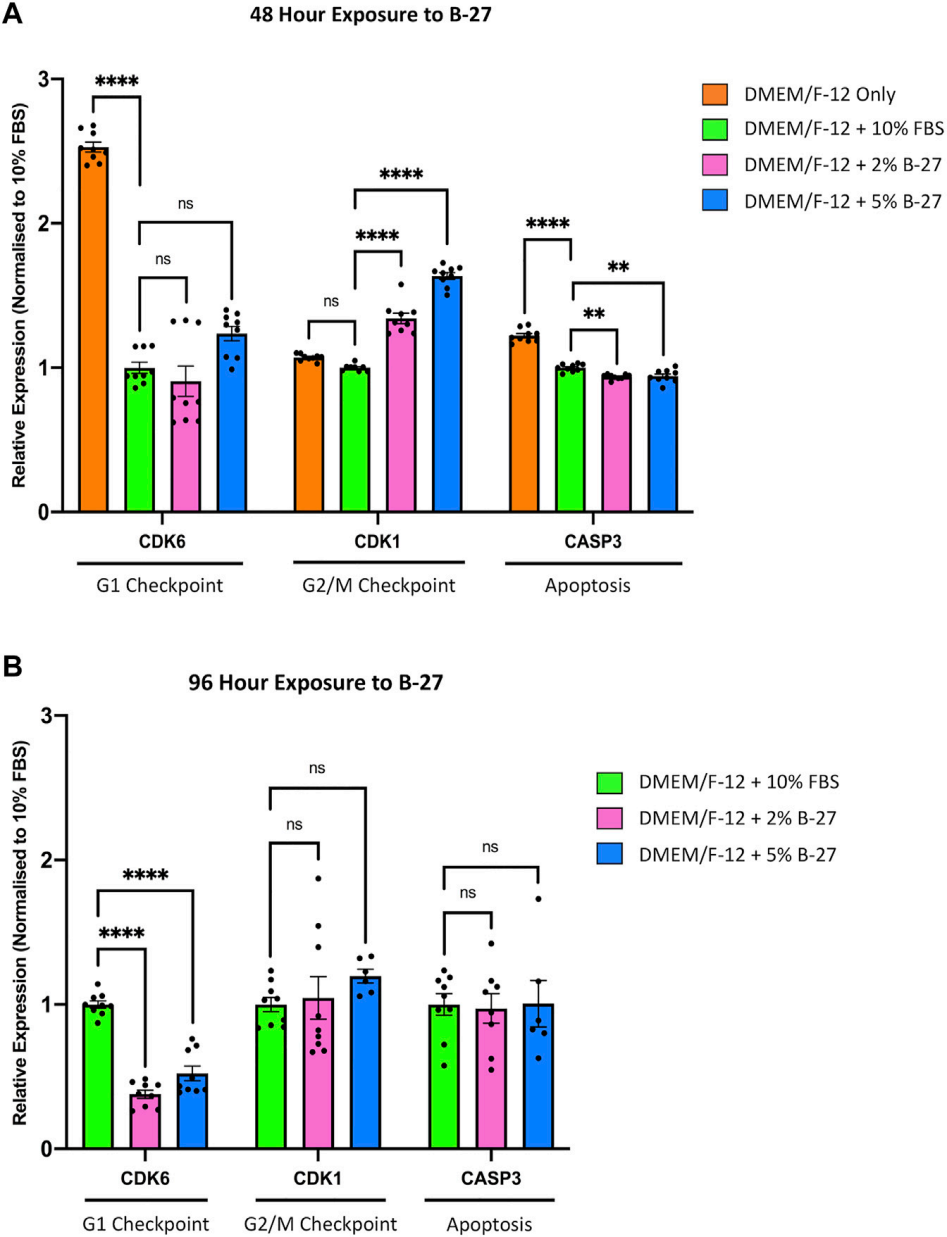
Longer term B-27 exposure leads to SH-SY5Y cell cycle arrest. SH-SY5Y cells were cultured in DMEM/F-12 only, or DMEM/F-12 supplemented with either 10% FBS, 2% B-27 or 5% B-27 for **(A)** 48 h or **(B)** 96 h. qRT-PCR was performed to measure mRNA expression of cell cycle markers *CDK6* and *CDK1*, and apoptosis marker *CASP3*. Relative expression of *CDK1*, *CDK6*, and *CASP3* (standardized to *GAPDH* and *POLR2A*) normalized to the DMEM/F-12 + 10% FBS culture conditions. Data presented as mean ± SEM. N = 3 independent biological samples with two to three technical replicates each. Statistical significance against the DMEM/F-12 + 10% FBS condition was determined using ordinary one-way ANOVA and Tukey’s multiple comparison tests in GraphPad Prism version 9.0.0 for Mac, GraphPad Software, San Diego, California, United States, www.graphpad.com. ns = not significant; *p < 0.05; **p < 0.01; ****p < 1 × 10^–4^. *CASP3, Caspase 3*; *CDK1, Cyclin dependent kinase* 1; *CDK6, Cyclin dependent kinase* 6; DMEM/F-12, Dulbecco’s modified eagle medium/nutrient mixture F-12 with GlutaMAX supplement; FBS, fetal bovine serum; *GAPDH, Glyceraldehyde-3-phosphate dehydrogenase*; mRNA, messenger RNA; *POLR2A*, *RNA polymerase II subunit A*; SEM, standard error of the mean.

To investigate the molecular mechanisms behind the observed reduction in proliferation between SH-SY5Y cells cultured in FBS and B-27 supplement, we performed qRT-PCR to measure the expression of cell cycle markers, *Cyclin-dependent kinase 6* (*CDK6*) (G1 checkpoint) and *Cyclin-dependent kinase 1 (CDK1)* (G2/M checkpoint). As a “negative control” we used SH-SY5Y cells cultured in DMEM/F-12 only. Under consistent serum starvation, SH-SY5Y cells show high levels of apoptosis and begin to differentiate (visible neurite formation), thus exiting the cell cycle. We observed no difference in *CDK6* expression between SH-SY5Y cells cultured in FBS or B-27 after 48 h (*p* > 0.05). On the other hand, *CDK1* expression was higher in the B-27-cultured cells compared to the FBS-cultured cells after 48 h (*p* < 1 × 10^–4^) (Figure 4A). This suggests that B-27 exposure may promote progression through the G2/M checkpoint, potentially accelerating entry into mitosis or increasing the responsiveness of these cells to mitogenic stimuli. In contrast, following longer 96-h exposure to B-27 (before the sudden change in SH-SY5Y proliferation), *CDK1* expression was no longer different between SH-SY5Y cells cultured in FBS or B-27 (*p* > 0.05), and instead *CDK6* expression was significantly lower in B-27-cultured SH-SY5Y cells compared to FBS-cultured cells (*p* < 1 × 10^–4^) (Figure 4B). This trend for falling *CDK6* and *CDK1* expression following longer-term exposure to B-27 suggests that the B-27-cultured SH-SY5Y cells are beginning to exit the cell cycle and entering a more quiescent, G0-like state. This agrees with the sudden reduced proliferative capacity observed from day 5 onwards in the growth curve experiments.

To ensure that the observed reduction in proliferation in B-27-cultured SH-SY5Y cells was not due to increased cell death, we measured the expression of the apoptosis marker *Caspase 3* (*CASP3*). As expected, we found that serum-starved SH-SY5Y cells cultured in DMEM/F-12 only exhibited significantly higher expression of *CASP3* compared to SH-SY5Y cells cultured in 10% FBS, as well as SH-SY5Y cells cultured in 2% and 5% B-27 (*p* > 1 × 10^–4^) (Figure 4A). Interestingly, SH-SY5Y cells exposed to B-27 for 48-h showed reduced *CASP3* expression compared to SH-SY5Y cells cultured in 10% FBS (*p* < 0.01) (Figure 4A). This suggests that short-term exposure to B-27 could protect SH-SY5Y cells from apoptosis. However, after 96-h, SH-SY5Y cells cultured in B-27 showed no difference in *CASP3* expression than SH-SY5Y cells cultured in FBS for 96 h (*p* > 0.05) (Figure 4B). Ultimately, these findings suggest that SH-SY5Y cells cultured in B-27 show no change in apoptosis compared to SH-SY5Y cells cultured in FBS, and that B-27 is clearly not toxic towards SH-SY5Y cells (as compared to serum starvation, which shows significantly higher *CASP3* expression (*p* < 1 × 10^–4^)].

### 3.4 B-27 induces SH-SY5Y cell differentiation and promotes neurite outgrowth

**FIGURE 5:**
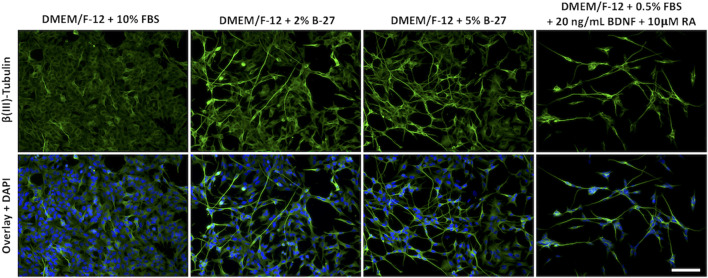
B-27 encourages neurite outgrowth and differentiation in SH-SY5Y cells. SH-SY5Y cells were cultured in DMEM/F-12 supplemented with either 10% FBS, 2% B-27 supplement, 5% B-27 supplement or 0.5% FBS + 20 ng/ml BDNF + 10 μM RA for 5 days. Cells were fixed with 4% PFA for 20 min. Microtubules were stained with 1:500 mouse anti-β(III)-Tubulin (*R&D Systems*, #MAB1195) and visualized with 1:400 Alexa Fluor^®^ 488-conjugated donkey anti-mouse (*Thermo Scientific*, #A21202) (green). Nuclei were counterstained with 1:5,000 DAPI (blue). Images taken at ×20 magnification through the DAPI channel (excitation 325–375 nm; emission 435–485 nm) and the FITC channel (excitation 460–500 nm; emission 512–542 nm) on the Leica DM4000 LED upright fluorescent microscope. Scale bar = 100 μm. BDNF, brain-derived neurotrophic factor; DAPI, 4′,6-Diamidino-2-phenylindole; DMEM/F-12, Dulbecco’s modified eagle medium/nutrient mixture F-12 with GlutaMAX supplement; FBS, fetal bovine serum; FITC, fluorescein isothiocyanate; PFA, Paraformaldehyde; RA, retinoic acid.

Although we were unable to successfully passage SH-SY5Y cells cultured in B-27 (Supplementary File 1), we noticed that SH-SY5Y cells exposed to B-27 exhibited a slightly more differentiated phenotype. Previous studies have shown that following differentiation into more neuron-like populations, SH-SY5Y cells are withdrawn from the cell cycle, entering a state of quiescence, and leading to reduced proliferation rates (Påhlman et al., 1984; Encinas et al., 2000; Mollereau et al., 2007; Cernaianu et al., 2008; Guarnieri et al., 2009; Kovalevich and Langford, 2013). We observed that SH-SY5Y cells cultured in B-27 proliferated at a slower rate compared to SH-SY5Y cells cultured in FBS (Figure 3) and demonstrated cell cycle arrest (Figure 4). Therefore, we assessed the morphology of SH-SY5Y cells cultured in B-27 compared to SH-SY5Y cells cultured in DMEM/F-12 + 10% FBS. We also compared B-27 exposed SH-SY5Y cells to SH-SY5Y cells cultured with RA and BDNF, factors previously demonstrated to induce SH-SY5Y cell differentiation (Kovalevich and Langford, 2013). SH-SY5Y cells were cultured for 5 days, fixed with 4% PFA and stained for β(III)-Tubulin (TUBB3) to visualize the microtubule network. We observed that SH-SY5Y cells cultured with 10 μM RA and 20 ng/ml BDNF under serum starvation displayed more pyramidal shaped cell bodies, extended long processes and visually increased TUBB3 immunolabeling intensity (Figure 5). On the other hand, SH-SY5Y cells cultured with 10% FBS appeared more epithelial-like with very few long processes and exhibited visually weaker TUBB3 immunolabeling intensity (Figure 5). Additionally, SH-SY5Y cultured with 10 μM RA and 20 ng/ml BDNF exhibited much higher levels of cell death than SH-SY5Y cells cultured with 10% FBS, with fewer cells surviving following 5 days of differentiation (Figure 5). Similar to those cultured with RA and BDNF, we observed that SH-SY5Y cells cultured in 2% and 5% B-27 exhibit more pyramidal cell bodies along with long processes (Figure 5). However, unlike those cells, B-27-cultured cells show much lower levels of cell death (Figure 5). These findings suggest that exposure to B-27 promotes neurite extension, and differentiation with B-27 leads to lower levels of cell death compared to typical SH-SY5Y differentiation protocols involving serum-starvation.

**FIGURE 6:**
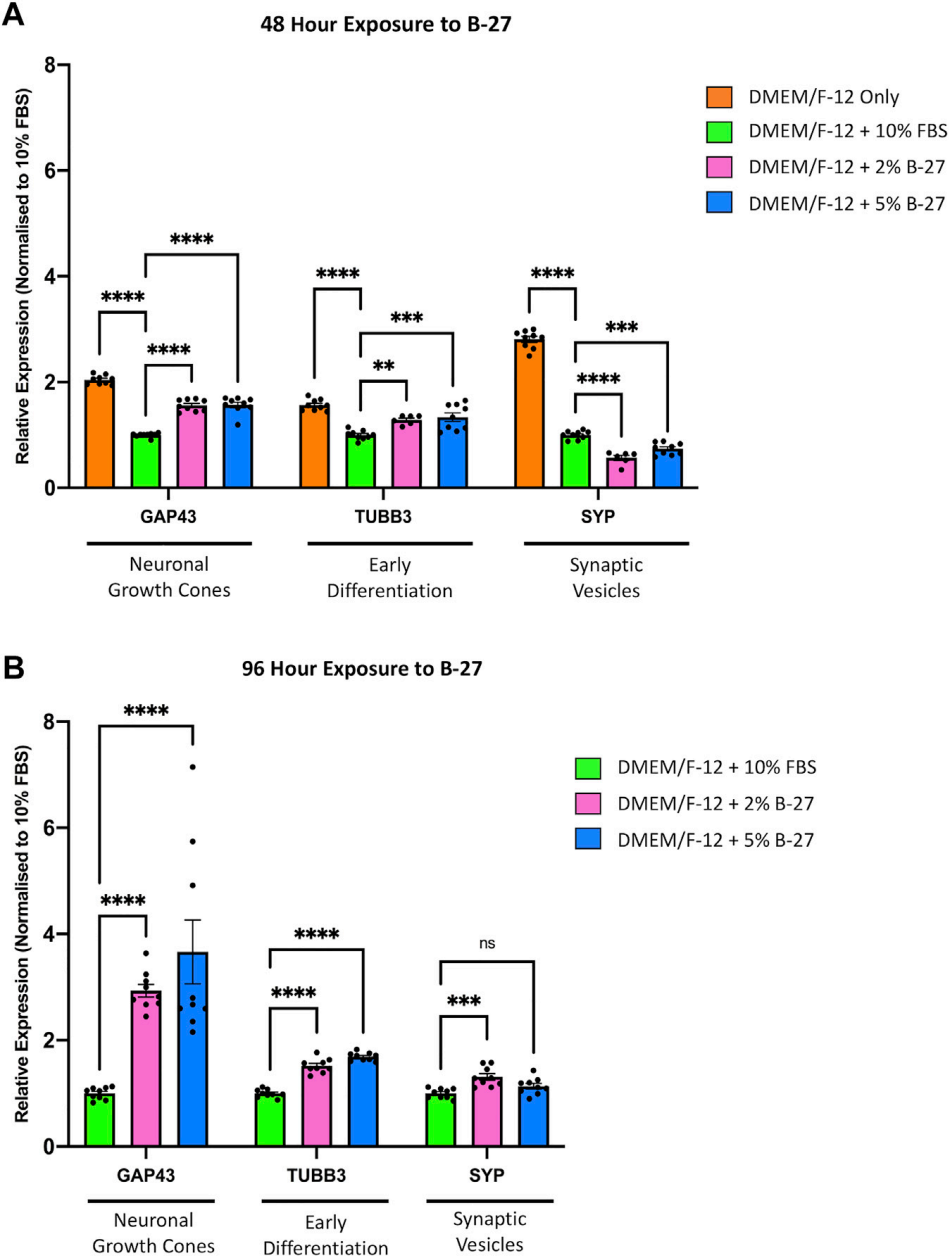
B-27 induces SH-SY5Y cell differentiation. SH-SY5Y cells were cultured in DMEM/F-12 only, or DMEM/F-12 supplemented with either 10% FBS, 2% B-27 or 5% B-27 for **(A)** 48 h or **(B)** 96 h. qRT-PCR was performed to measure mRNA expression of differentiation markers, *GAP43*, *TUBB3* and *SYP*. Relative expression of *GAP43*, *TUBB3*, and *SYP* (standardized to *GAPDH* and *POLR2A*) normalized to the DMEM/F-12 + 10% FBS culture conditions. Data presented as mean ± SEM. N = 3 independent biological samples with two to three technical replicates each. Statistical significance against the DMEM/F-12 + 10% FBS condition was determined using ordinary one-way ANOVA and Tukey’s multiple comparison tests in GraphPad Prism version 9.0.0 for Mac, GraphPad Software, San Diego, California, United States, www.graphpad.com. ns = not significant; *p < 0.05; **p < 0.01; ***p < 1 × 10^–3^; ****p < 1 × 10^–4^. DMEM/F-12, Dulbecco’s modified eagle medium/nutrient mixture F-12 with GlutaMAX supplement; FBS, fetal bovine serum; *GAP43*, *Growth associated protein 43*; *GAPDH, Glyceraldehyde-3-phosphate dehydrogenase*; mRNA, messenger RNA; *POLR2A*, *RNA polymerase II subunit A*; SEM, standard error of the mean; *SYP*, *Synaptophysin*; *TUBB3, Tubulin beta 3 class III*.

To further investigate whether exposure to B-27 can promote differentiation of SH-SY5Y cells into more mature neuron-like cells, we performed qRT-PCR to measure the expression of neuronal differentiation markers, *Growth associated protein 43* (*GAP43*), *TUBB3*, and *Synaptophysin* (*SYP*), which have previously been demonstrated to be upregulated in SH-SY5Y cells differentiated with various differentiation-promoting factors (Dwane et al., 2013; Kovalevich and Langford, 2013). We observed that serum-starved SH-SY5Y cells exhibited significantly higher *GAP43*, *TUBB3*, and *SYP* expression compared to SH-SY5Y cells cultured with DMEM/F-12 + 10% FBS (*p* < 1 × 10^–4^) (Figure 6A). This confirms previous studies that suggest serum starvation promotes SH-SY5Y cell differentiation. We also observed that SH-SY5Y cells cultured in 2% and 5% B-27 for 48 h showed increased expression of *GAP43* (*p* < 1 × 10^–4^) and *TUBB3* (*p* < 0.01 and *p* < 1 × 10^–3^, respectively) compared to SH-SY5Y cells cultured in 10% FBS for 48 h (Figure 6A). Moreover, the expression of *GAP43* and *TUBB3* was further increased in SH-SY5Y cells cultured with B-27 for 96 h, compared to SH-SY5Y cells cultured with FBS for 96 h (*p* < 1 × 10^−4^) (Figure 6B). This suggests that early exposure to B-27 promotes SH-SY5Y differentiation into a more neuron-like state, and longer-term exposure to B-27 not only maintains but also further promotes this differentiation. In contrast to the serum-starvation, B-27-cultured SH-SY5Y cells show initially reduced *SYP* expression compared to the FBS control (*p* < 1 × 10^–4^ and *p* < 1 × 10^−3^, respectively) (Figure 6A). This suggests that although B-27 exposure may initiate neuritogenesis, it may downregulate or impede the formation of mature synapses. However, following longer-term exposure, B-27-cultured SH-SY5Y cells begin to show a trending increase in *SYP* expression compared to the FBS-cultured SH-SY5Y cells (*p* < 1 × 10^−3^) (Figure 6B).

### 3.5 B-27 is equally effective at promoting SH-SY5Y cell neurite extension as BDNF and serum starvation

**FIGURE 7:**
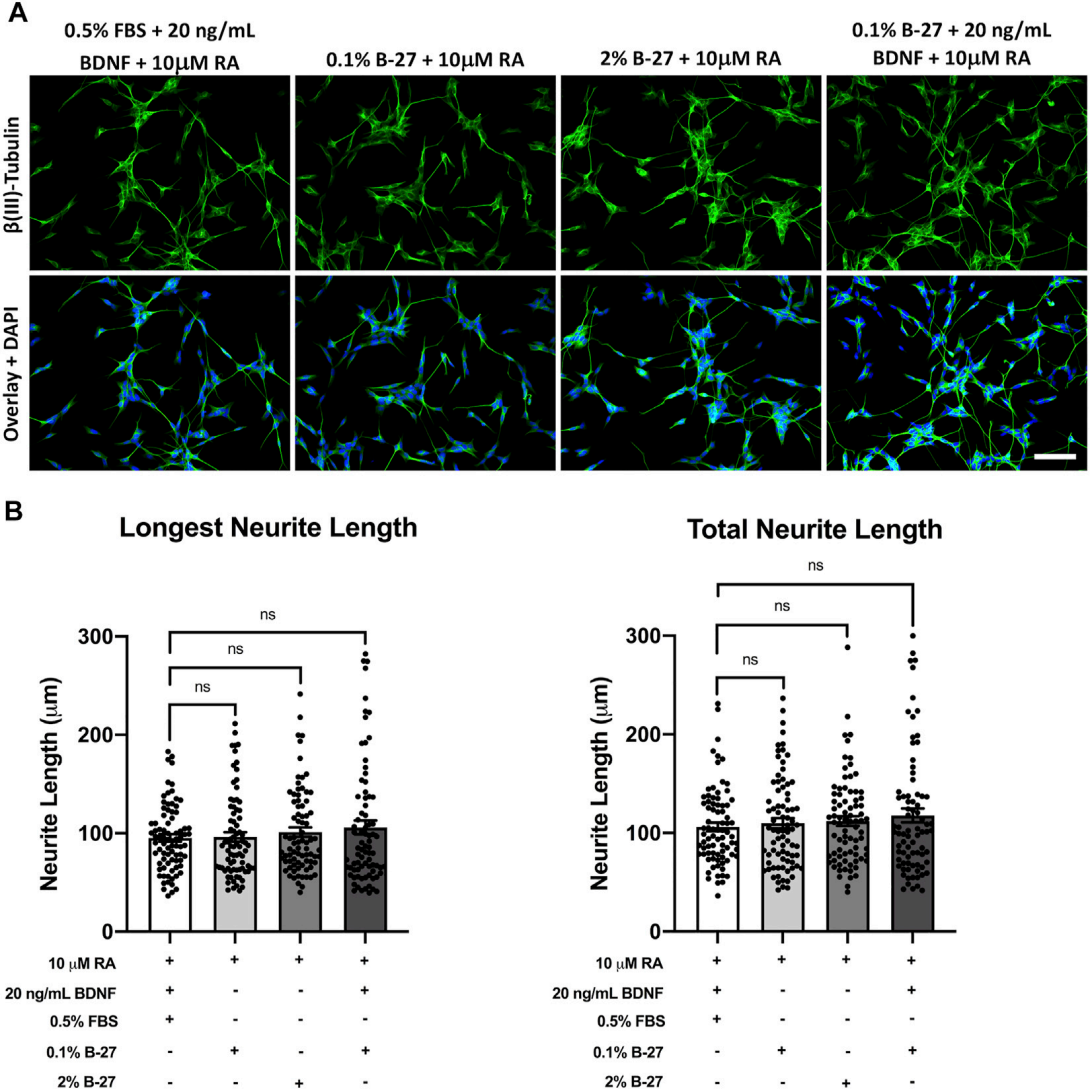
SH-SY5Y cells differentiated with B-27 show no significant difference in neurite length compared with SH-SY5Y cells differentiated with BDNF. **(A)** SH-SY5Y cells were differentiated for 5 days with DMEM/F-12 + 10 μM RA, in conjunction with varying combinations of neurite outgrowth-stimulating molecules (B-27 or BDNF) or serum starvation (0.5% FBS). Cells were fixed with 4% PFA for 20 min. Microtubules were stained with 1:500 mouse anti-β(III)-Tubulin (*R&D Systems*, #MAB1195) and visualized with 1:400 Alexa Fluor^®^ 488-conjugated donkey anti-mouse (*Thermo Scientific*, #A21202) (green). Nuclei were counterstained with 1:5,000 DAPI (blue). Images taken at ×20 magnification. Scale bar = 100 μm. **(B)** Longest neurite length and total neurite length measurements were taken using the *FIJI* software (Schindelin et al., 2012). Three independent experimental replicates were performed and 80 cells per condition were measured in total. Data presented as mean ± SEM. Statistical significance against the DMEM/F-12 + 10% FBS condition was determined using ordinary one-way ANOVA and Tukey’s multiple comparison tests in GraphPad Prism version 9.0.0 for Mac, GraphPad Software, San Diego, California, United States, www.graphpad.com. ns = not significant. BDNF, brain-derived neurotrophic factor; DAPI, 4′,6-Diamidino-2-phenylindole; DMEM/F-12, Dulbecco’s modified eagle medium/nutrient mixture F-12 with GlutaMAX supplement; FBS, fetal bovine serum; FITC, fluorescein isothiocyanate; PFA, Paraformaldehyde; RA, retinoic acid.

Having shown that B-27 promotes SH-SY5Y cell neurite outgrowth, we sought to determine whether B-27 is as effective at inducing neurite extension as the typical SH-SY5Y differentiation-promoting factors, and whether combining B-27 with these factors could further promote neurite extension. Therefore, we cultured SH-SY5Y cells in DMEM/F-12 with varying combinations of FBS, B-27, RA and BDNF for 5 days, and performed neurite tracing analysis. A variety of media compositions were used: 1) serum starvation with RA and BDNF, the “typical” SH-SY5Y differentiation protocol; 2) RA with low concentrations of B-27; 3) RA with 2% B-27; and 4) RA with low B-27 and BDNF. RA was included in all cases as a common denominator differentiation-promoting molecule. As typical SH-SY5Y cell differentiation protocols use serum-starvation to induce differentiation, low concentrations of B-27 (0.1%) were used in this experiment to test whether a similar fold-reduction in B-27 to FBS could induce effective SH-SY5Y cell differentiation. We found there was no significant difference in longest neurite length or total neurite length between SH-SY5Y cells cultured in the above media compositions (*p* > 0.05) (Figure 7). This indicates that RA and BDNF are very effective at inducing SH-SY5Y cell differentiation, and that adding B-27 does not further improve the effectiveness of RA and BDNF at promoting neurite extension.

### 3.6 SH-SY5Y cells differentiate into a more glutamatergic-like phenotype when cultured with B-27

**FIGURE 8:**
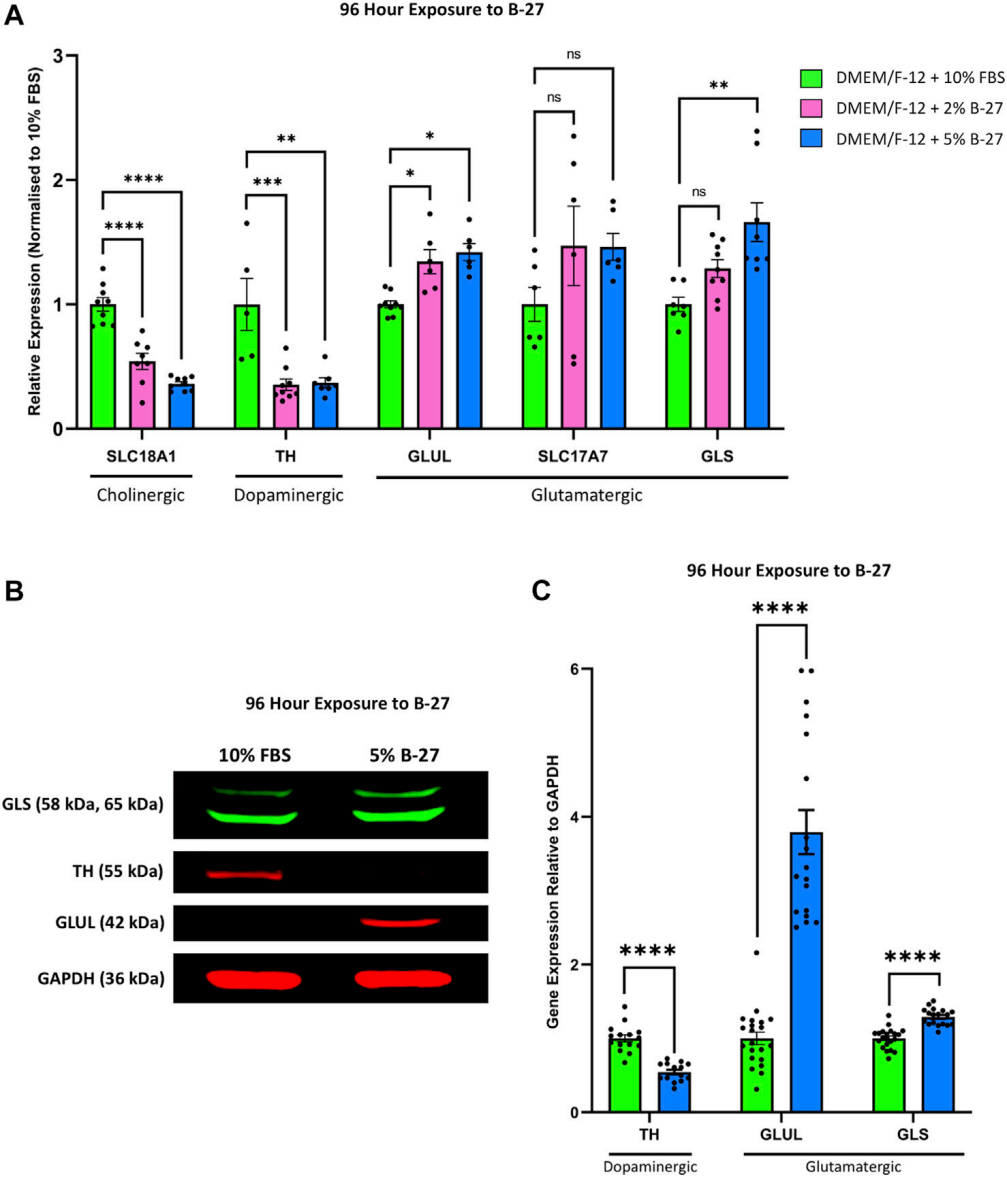
SH-SY5Y cells differentiate into a more glutamatergic-like phenotype following treatment with B-27. **(A)** SH-SY5Y cells were cultured in DMEM/F-12 supplemented with either 10% FBS, 2% B-27 or 5% B-27 for 96 h. qRT-PCR was performed to measure mRNA expression of cholinergic marker *SLC18A1* (VMAT1), dopaminergic marker *TH* and glutamatergic markers *GLUL*, *SLC17A7* (VGLUT1) and *GLS*. Relative expression of *SLC18A1, TH, GLUL, SLC17A7* and *GLS* (standardized to *GAPDH* and *POLR2A*) normalized to the DMEM/F-12 + 10% FBS culture conditions. Data presented as mean ± SEM; N = 3 independent biological samples with two to three technical replicates each. Statistical significance was determined using ordinary one-way ANOVA and Tukey’s multiple comparison tests in GraphPad Prism version 9.0.0 for Mac, GraphPad Software, San Diego, California, United States, www.graphpad.com. ns = not significant; *p < 0.05; **p < 0.01; ***p < 1 × 10^–3^; ****p < 1 × 10^–4^. **(B)** Western blot of lysate from SH-SY5Y cells cultured in DMEM/F-12 supplemented with either 10% FBS or 5% B-27 for 96 h. Membranes were probed with rabbit anti-GLS (*Proteintech* #29519-1-AP), mouse anti-TH (*Proteintech*, #66334-1-Ig), mouse anti-GLUL (*Proteintech*, #66323-1-Ig) and mouse anti-GAPDH (*Santa Cruz Biotechnology*, #sc-47724). GAPDH was used as a loading control. Molecular weights are as follows GLS = 58 kDa and 65 kDa; TH = 55 kDa; GLUL = 42 kDa; GAPDH = 36 kDa. **(C)** Levels of protein expression relative to GAPDH were quantify through densitometric analysis using the *FIJI* software (Schindelin et al., 2012). Three independent experiments were performed and densitometric analysis performed using seven independent blots. Data presented as mean ± SEM. Statistical significance against the DMEM/F-12 + 10% FBS condition was determined using Unpaired t tests in GraphPad Prism version 9.0.0 for Mac, GraphPad Software, San Diego, California, United States, www.graphpad.com. ****p < 1 × 10^–4^. DMEM/F-12, Dulbecco’s modified eagle medium/nutrient mixture F-12 with GlutaMAX supplement; FBS, fetal bovine serum; *GAPDH, Glyceraldehyde-3-phosphate dehydrogenase*; *GLS, Glutaminase*; *GLUL, Glutamate-ammonia ligase*; mRNA: messenger RNA; *POLR2A, RNA polymerase II subunit A*; SEM, standard error of the mean; *SLC17A7, Solute carrier family 17 member 7*; *SLC18A1, Solute carrier family 18 member A1; TH, tyrosine hydroxylase*; *VGLUT1, vesicular glutamate transporter 1*; *VMAT1, Vesicular monoamine transporter 1*.

Previous studies have demonstrated that SH-SY5Y cells can be differentiated towards a number of neuronal phenotypes including cholinergic, dopaminergic and adrenergic, and that this is dependent on the media conditions used (Kovalevich and Langford, 2013). As such, we decided to assess the phenotype of SH-SY5Y cells differentiated with B-27 by qRT-PCR. We found that SH-SY5Y cells cultured in 2% and 5% B-27 for 96 h showed significantly lower expression of the cholinergic neuronal marker *Solute carrier family 18 member 1* (*SLC18A1*) (encodes for VMAT1) (*p* < 1 × 10^–4^), and significantly lower expression of the dopaminergic neuronal marker *TH* (*p* < 1 × 10^–3^ and *p* < 0.01, respectively) compared to SH-SY5Y cells cultured in 10% FBS (Figure 8A). This suggests that exposure to B-27 does not drive SH-SY5Y cells towards a cholinergic or dopaminergic phenotype. We also measured the mRNA expression of several glutamatergic neuronal markers, including *Glutamate-ammonia ligase* (*GLUL*), *Solute carrier family 17 member* (*SLC17A7*) [encodes for Vesicular glutamate transporter 1 (VGLUT1)] and *Glutaminase* (*GLS*). *GLUL* expression was significantly upregulated in B-27-cultured SH-SY5Y cells compared to FBS-cultured SH-SY5Y cells (*p* < 0.05) (Figure 8A). Additionally, there was also a trend for increased expression of *SLC17A7* and *GLS* in SH-SY5Y cells cultured in B-27 compared to SH-SY5Y cells cultured in 10% FBS, however this increase was only significant when comparing *GLS* expression between SH-SY5Y cells cultured in 5% B-27 and SH-SY5Y cells cultured in 10% FBS (*p* < 0.01) (Figure 8A). These findings suggest that SH-SY5Y cells differentiate towards a more glutamatergic-like phenotype as opposed to a more cholinergic-like or dopaminergic-like phenotype, following long-term exposure to B-27.

Following on from this, we performed Western blotting to confirm whether these changes in mRNA expression carried through to the protein level. 5% B-27 was chosen for these experiments as we found in our qRT-PCR experiments that SH-SY5Y cells cultured in DMEM/F-12 + 5% B-27 exhibited slightly higher levels of *GLUL* and *GLS* expression than those cultured in 2% B-27. In agreement with our qRT-PCR data, we observed that SH-SY5Y cells cultured in 5% B-27 for 96 h showed a significant reduction in TH expression compared to SH-SY5Y cells cultured in 10% FBS (*p* < 1 × 10^–4^) (Figures 8B,C). Moreover, GLUL and GLS expression was significantly greater in B-27-cultured cells compared to FBS-cultured cells (*p* < 1 × 10^–4^) (Figures 8B,C). These findings suggest that long-term exposure to B-27 drives SH-SY5Y cells to differentiate towards a more glutamatergic phenotype, characterized by increased mRNA and protein expression of glutamatergic markers.

## 4 Discussion

### 4.1 B-27 induces cell cycle arrest through reduced CDK6 expression

Our study outlines the potential for B-27 in SH-SY5Y cell culture. Initially, B-27 performed similarly to FBS, encouraging SH-SY5Y proliferation (Figure 2). However, subsequent growth curve and Ki-67 staining analyses revealed that SH-SY5Y cells exhibit reduced proliferative capacity after longer-term (5 days) exposure to B-27 (Figure 3). B-27-cultured SH-SY5Y cells initially show comparable doubling and numbers of actively proliferating cells to FBS-cultured SH-SY5Y cells, yet after 4 days (96 h) of B-27 exposure this trend stops, and cell proliferation is largely reduced compared to FBS-cultured SH-SY5Y cells. This is supported by our qRT-PCR experiments measuring *CDK6* (G1 checkpoint) expression. Upon initial 48-h exposure to B-27, *CDK6* expression is unchanged between SH-SY5Y cells cultured in FBS and B-27 (Figure 4A). However, following 96-h of B-27 exposure, *CDK6* expression is largely reduced (Figure 4B). Rising levels of CDK6 are reported to mediate the exit of stem cells from a state of quiescence into cell cycling following mitogenic stimulation (Laurenti et al., 2015). Moreover, several studies have demonstrated that CDK6 activity prevents G1 phase arrest and stops cells from returning to a quiescence state before entering the S phase (Yang et al., 2020), thereby promoting continuation of the cell cycle (Lin et al., 2001; Kim et al., 2002). As such, the observed reduction in *CDK6* expression after 96-h exposure may indicate that B-27-cultured SH-SY5Y cells are entering a quiescent state, showing limited progression through the G1 phase of the cell cycle. This could also explain the sudden attenuation in increasing cell number in the subsequent days of the growth curve experiments, with the B-27-cultured SH-SY5Y cells exhibiting a 4-day doubling time as opposed to the 1-day doubling time observed in the FBS-cultured SH-SY5Y cells from day 5 onwards, and the marked reduction in the percentage of actively proliferating cells in the B-27 condition (as measured by the proportion of Ki-67+ cells).

However, SH-SY5Y cells cultured in DMEM/F-12 show high *CDK6* expression relative to the FBS-cultured SH-SY5Y cells (Figure 4A). Upon serum starvation, cells initially enter a reversible G0 state in which reintroduction of serum leads to cell cycle re-entry (Urbaìn and Cheung, 2021). Following prolonged periods of serum starvation, cells can enter an irreversible G0 state (Urbaìn and Cheung, 2021). As CDK6 has been reported to mediate resistance to cell cycle arrest in response to serum starvation in some cell lines (Lin et al., 2001; Kim et al., 2002), this could be interpreted as a compensatory mechanism whereby the SH-SY5Y cells upregulate *CDK6* expression to counteract serum starvation. The exact mechanisms by which serum starvation induces increased *CDK6* expression, and the consequence of this will need further investigation.

### 4.2 Effects of B-27 on SH-SY5Y cell mitosis, cell adhesion and apoptosis

In addition to cell cycle progression, we also aimed to investigate if B-27 could alter the balance of cell division and death in SH-SY5Y populations. To accomplish this, we measured the expression of *CDK1* (regulator of the G2/M phase transition) and *CASP3* (apoptotic marker). CDK1 is an important regulator of mitosis, and its stable activity ensures G2/M phase progression in neural stem and progenitor cells (Jiang and Hsieh, 2014). After initial 48-h exposure to B-27, *CDK1* expression is elevated and *CASP3* expression reduced compared to the FBS control (Figure 4A). Importantly, SH-SY5Y cells cultured in DMEM/F-12 only (our “positive control” for increased apoptosis) show highest *CASP3* expression (Figure 4A). This demonstrates that B-27 is not toxic as a supplement for SH-SY5Y culture. Moreover, the increased *CDK1* expression in B-27-cultured SH-SY5Y cells potentially suggests increased mitotic rate. However, our growth curve does not show quicker doubling times, nor does our Ki-67 staining analysis show an increase in the percentage of proliferating SH-SY5Y cells in the B-27-cultured condition (Figure 3), suggesting mitotic rate is not increased. Recently, CDK1 has been reported to regulate cell adhesion during cell division (Jones et al., 2018). Before mitosis, cells have to disassemble adhesion complexes and retract from the extracellular matrix (Jones et al., 2018). Inactivation of CDK1-cyclin complexes triggers this retraction from the extracellular matrix and allows for mitotic entry (Jones et al., 2018). Interestingly, we observed that B-27 may alter cell attachment or adhesion (Supplementary Figure 1). Although this will require further investigation, perhaps excessive CDK1 activity in B-27-cultured SH-SY5Y cells could prevent mitotic entry due to a lack of retraction from the extracellular matrix or lead to impaired reattachment to the extracellular matrix or plate surface following cell division. Both reasons could explain the attenuated cell number increase observed in the growth curve experiments.

Whereas *CDK1* expression is initially elevated in B-27-cultured SH-SY5Y cells (relative to the FBS control) at the 48-h exposure time point, its expression reduces to become equivalent to the FBS control at the 96-h time point (Figure 4B). This indicates a trend for decreasing *CDK1* expression in SH-SY5Y cells following longer periods of culture in B-27. Agreeing with this, CDK1 expression was previously reported to be reduced in quiescent SH-SY5Y cells (Hiyoshi et al., 2012). Therefore, in line with the previously discussed *CDK6* downregulation, a trend for decreasing *CDK1* expression also indicates SH-SY5Y cell entry into a quiescent state following longer periods of exposure to B-27. Although not tested in this study, it would be interesting to see if *CDK1* expression is further reduced in SH-SY5Y cells cultured in B-27 for prolonged periods (9 days or longer). This would help to confirm that B-27 exposure shifts the SH-SY5Y cell cycle, leading to cell cycle arrest. Notably, there is no difference in *CASP3* expression at the 96-h time point (Figure 4B), which suggests that the reduced proliferation we see from day 5 onwards in the growth curve experiments is not likely due to increased rates of apoptosis. This also reinforces that B-27 is not toxic towards SH-SY5Y cells.

### 4.3 B-27 promotes expression of neuronal differentiation markers

Previous studies have demonstrated that SH-SY5Y cells enter a state of quiescence following differentiation into a more mature neuron-like state (Ferguson and Subramanian, 2016). We observed that SH-SY5Y cells cultured in B-27 similarly show limited progression through the cell cycle and entry into quiescence. Therefore, we decided to investigate whether B-27 induces cell cycle exit leading to differentiation, so we measured the expression of multiple genes known to be upregulated in differentiated SH-SY5Y cells (*GAP43*, *TUBB3*, and *SYP*) (Sarkanen et al., 2007; Dwane et al., 2013; Shipley et al., 2016). GAP43 is a good marker for neuritogenesis and neurite extension, as it is highly expressed in growth cones (Nozumi et al., 2009; Kawasaki et al., 2018). TUBB3 is a marker for immature neurons (Menezes and Luskin, 1994) and its expression is also increased in mature neurons, largely attributed to the extensive microtubule network within axons and dendrites (Lasser et al., 2018). SYP is an important synaptic component that regulates the endocytosis and formation of synaptic vesicles (Tarsa and Goda, 2002; Pyeon and Lee, 2012). We observed increased mRNA expression of *GAP43* and *TUBB3* following short-term (48-h) and longer-term (96-h) exposure to B-27 compared to the FBS control (Figures 6A,B). Visually, we noticed that SH-SY5Y cells cultured in B-27 began to extend and form networks of neuritic processes, showing strong TUBB3 staining intensity comparable to that of SH-SY5Y cells cultured in the differentiation-promoting factors, RA and BDNF (Figure 5). These changes in cell morphology complement and further validate the increased mRNA expression of *GAP43* and *TUBB3* observed in our qRT-PCR experiments, suggesting that B-27 promotes SH-SY5Y cell differentiation. Importantly, increased CDK6 activity has been reported to inhibit cellular differentiation (Matushansky et al., 2003; Grossel and Hinds, 2006; Fujimoto et al., 2007), and *CDK6* expression has also been observed to decrease over time as neural stem cells differentiate (Ferguson et al., 2000; Choe et al., 2010). Together with the observed reduction in *CDK6* expression in B-27-cultured SH-SY5Y cells, these findings support that long-term B-27 exposure induces cell cycle exit towards a quiescent state before SH-SY5Y cells differentiate towards a more neuronal state.

Whether B-27 stimulates the formation of mature synapses in SH-SY5Y cells is harder to determine. Initially, we found that *SYP* expression is reduced at the 48-h time point compared to FBS-cultured SH-SY5Y cells (Figure 6A), potentially indicating that although B-27 is effective in inducing neuritogenesis, the differentiated SH-SY5Y cells produced through this process may be more synaptically immature. However, we saw that *SYP* expression increased to become slightly higher than the FBS control at the 96-h time point (Figure 6B). Previous studies report increased SYP expression in SH-SY5Y cells following short-term (Da Costa et al., 2021) and longer-term exposure to RA (Sarkanen et al., 2007; Cheung et al., 2009). This suggests that whilst initial exposure to B-27 may induce neurite outgrowth, SH-SY5Y cells only begin to adopt a more mature neuron-like phenotype following 96 h of exposure to B-27. Furthermore, in contrast to RA, which is a potent and rapid inducer of differentiation, B-27 is subtler and requires longer to produce differentiated neuron-like SH-SY5Y cells. It is likely that *SYP* expression would continue to increase over time in B-27-cultured SH-SY5Y cells, however, further experiments will be required to determine whether SH-SY5Y cells cultured in B-27 for prolonged periods (e.g., 2 weeks or longer) form functional synapses.

Altogether, the increased *GAP43*, *TUBB3* and *SYP* expression, combined with the reduced *CDK6* expression present strong evidence that SH-SY5Y cells become more neuron-like following 96 h of exposure to B-27. However, it is unclear how B-27 causes this switch towards a quiescent or G0-like differentiation state. Increased *GAP43* and *TUBB3* expression at the 48-h time point suggests that B-27 quite early on initiates activation of molecular pathways regulating neuronal differentiation. However, the fact that *CDK6* expression and cell proliferation is reduced only after longer 96-h exposure implies that SH-SY5Y cells may not fully commit to cell cycle exit before this point. As such, the increased expression of differentiation markers preludes the actual entry into a more mature “neuron-like” state. This may be an important distinction to make in experiments utilizing “differentiated” SH-SY5Y cells, as increased expression of the differentiation markers does not mean the SH-SY5Y cells have become “neurons”. Interestingly though, the fact that the B-27-cultured SH-SY5Y cell number continues to increase past day five implies that B-27 produces a heterogeneous population in which not all SH-SY5Y cells are differentiated. This is supported by increase in Ki-67+ SH-SY5Y cells after 8 days of exposure to B-27, which suggests either that a large proportion of SH-SY5Y cells appear to re-enter the cell cycle, or perhaps that the remaining proliferating cells from Day 5 have continued to divide and have “taken over” the culture. This could be attributed to differences in how S- and N-type SH-SY5Y cells respond to B-27. We can also see that some B-27-cultured SH-SY5Y cells in Figure 5 show a more epithelial S-type morphology and weaker TUBB3 staining. Therefore, it is possible that the N-type SH-SY5Y cells experience growth arrest, whereas the S-type SH-SY5Y cells continue to proliferate in B-27-supplemented media. Further experiments to characterize the heterogeneity of the B-27-cultured SH-SY5Y population could pave the way for generating purer differentiated SH-SY5Y cultures.

### 4.4 Application of B-27 promotes neurite outgrowth in SH-SY5Y differentiation to the same extent as serum starvation and BDNF

Applying B-27 for SH-SY5Y cell differentiation has previously been suggested to enhance differentiation (Kovalevich and Langford, 2013), so we sought to determine whether B-27 could enhance neurite extension when used in conjunction with RA, a strong SH-SY5Y cell differentiation factor. Serum starvation and BDNF are also commonly used in combination with RA to induce SH-SY5Y cell differentiation (Kovalevich and Langford, 2013). Serum starvation serves two roles—it not only helps to induce neurite formation, but it also prevents overgrowth of the S-type SH-SY5Y cells (Kovalevich and Langford, 2013; Shipley et al., 2016). BDNF promotes SH-SY5Y differentiation as well as survival of the N-type neuron-like SH-SY5Y cells whilst inhibiting replication of S-type SH-SY5Y cells (Kovalevich and Langford, 2013). As such, we used SH-SY5Y cells differentiated with RA and BDNF under serum starvation conditions as a “positive control” for neurite outgrowth, and we tested the effects of varying concentrations of B-27 with and without BDNF on SH-SY5Y cell neurite extension (Figures 7A,B). Although there was a slight trend for increasing longest neurite length and total neurite length when B-27 was implemented as part of the differentiation process, the difference compared to the typical SH-SY5Y differentiation protocol was very small and not statistically significant. This indicates that RA is quite likely already a potent inducer of SH-SY5Y differentiation, so the addition or combination of other factors (B-27, BDNF or serum starvation) for neurite outgrowth measurements has little impact on neurite length and is thus up to individual researcher choice of the type of differentiated SH-SY5Y “neurons” they aim to produce (e.g., dopaminergic, cholinergic, etc.).

However, the advantage of using B-27 for differentiation is that it is less toxic than serum starvation, as can be seen in Figure 5. Maintaining SH-SY5Y cells without serum for long periods can be quite difficult due to the high amount of apoptosis. Additionally, if SH-SY5Y cultures are mishandled, there is a risk of overgrowth of the S-type SH-SY5Y cells during prior to differentiation (Kovalevich and Langford, 2013; Shipley et al., 2016). This leads to disproportionate amounts of cell death during gradual serum starvation (Shipley et al., 2016). If larger numbers of differentiated SH-SY5Y cells are required, and if researchers plan to differentiate SH-SY5Y cells for long periods of time (e.g., 2 weeks or longer), using B-27 may be more suitable than typical differentiation protocols. We observed that SH-SY5Y cells continue to proliferate for a few days following exposure to B-27 before differentiating (Figure 3), and B-27-cultured SH-SY5Y cells do not show high levels of apoptosis or *CASP3* expression (Figure 4). For these reasons, B-27 could be useful to generate high yields of differentiated SH-SY5Y cells for high throughput and large-scale toxicity or pharmacological studies.

### 4.5 B-27 may induce SH-SY5Y differentiation towards a glutamatergic phenotype

SH-SY5Y cells are capable of differentiating into a variety of neuronal phenotypes depending on the factors used in their differentiation (Kovalevich and Langford, 2013). As we have previously demonstrated that B-27 causes SH-SY5Y differentiation, we wanted to determine the phenotype of the B-27-differentiated SH-SY5Y cells. Interestingly, we found that the expression of cholinergic (*SLC18A1*) and dopaminergic (*TH*) markers, known to be upregulated in differentiated SH-SY5Y cells (Kovalevich and Langford, 2013), are reduced in SH-SY5Y cells cultured in B-27 (Figure 8). Instead, we observed an increase in the expression of multiple glutamatergic markers (*GLUL*, *SLC17A7* and *GLS*). This indicates that the SH-SY5Y cells cultured in B-27 may be differentiating towards a glutamatergic phenotype.

We suspect that the expression of these glutamatergic markers may continue to increase following longer-term culture (e.g., 2 weeks or longer) in B-27, however that was not tested in this study. In fact, since the B-27-cultured SH-SY5Y cells did not appear to exit the cell cycle until 4–5 days of exposure, it can be argued that this was not sufficient time to allow for expression of mature glutamatergic markers. We also tried to measure the expression of other cholinergic markers (e.g., *CHAT*) and dopaminergic markers (e.g., *DAT*) to confirm our findings, however struggled to measure the expression of these genes in SH-SY5Y cells. We suspect that this was because these genes were too lowly expressed after 4-day culture in B-27 without additional SH-SY5Y differentiation-promoting factors. It would therefore be interesting for future studies to confirm this glutamatergic phenotype in SH-SY5Y cells differentiated with B-27 for much longer time periods and seeing if these glutamatergic SH-SY5Y “neurons” form mature synapses capable of secreting glutamate. For this reason, further experiments should be conducted in the future to characterize synaptic activity.

Overall, this B-27/SH-SY5Y culture system may serve as a valuable and cost-effective model of glutamatergic cells. The ease of SH-SY5Y cell culture makes this system particularly useful for preliminary studies or larger-scale toxicity screening studies where high cell numbers are required. In the future, this B-27/SH-SY5Y culture system can therefore be used to study many neurodegenerative and psychiatric diseases focusing on glutamatergic cells, and has the advantage of being directly translational due to the absence of any animal-derived components.

## 5 Conclusion and future directions

Our findings demonstrate that B-27 can support the production and survival of large numbers of differentiated SH-SY5Y cells with a glutamatergic phenotype. This opens up exciting avenues for research into the role of glutamatergic signaling in the biology of neurological disorders such as Autism spectrum disorder, Schizophrenia and Alzheimer’s disease. Although SH-SY5Y cells cannot completely replace the use or impact of primary or iPSC-derived neurons, they can still offer important perspectives on the molecular mechanisms underlying key neuronal functions. Importantly, SH-SY5Y cells are much more cost-effective to work with, making them invaluable for preliminary studies. SH-SY5Y cells are also easier to genetically modify than animal models or primary neurons, so this B-27/SH-SY5Y model provides an excellent system for investigating the impact of disease candidate genes on glutamatergic neurobiology.

Important questions still remain however, and further research is required to dissect the molecular mechanisms by which B-27 induces glutamatergic SH-SY5Y cell differentiation. Moreover, further research is required to fully characterize the phenotype of long-term B-27-differentiated SH-SY5Y cells. Future studies should see how far the B-27 SH-SY5Y system can go, testing to see if it can maintain SH-SY5Y cells for long periods of time (3 weeks or over), and if these cells continue to differentiate and/or proliferate over this period of time. This timing is quite important as current protocols to produce iPSC-derived neurons can take multiple weeks to fully establish mature glutamatergic neurons. Time-course comparisons with human neurons would therefore be necessary. SH-SY5Y cells are likely more similar to neural progenitor cells, expressing markers such as VIM, ASCL1 and STMN1 (Byrne et al., 2013; Ruangjaroon et al., 2017; Zammit et al., 2018), and as such, 1 week of culture in B-27 (as tested in this study) may not be sufficient to produce populations of mature glutamatergic SH-SY5Y “neurons”. Imaging and electrophysiological studies will also need to be performed to investigate whether these B-27 glutamatergic SH-SY5Y cells are synaptically mature and behave similarly to primary or stem cell-derived neurons. Addressing these gaps in our knowledge are important before this B-27/SH-SY5Y culture system can be used as a model for glutamatergic neurons in pharmacological or toxicity studies.

Importantly, we tested the use of a xeno-free formulation of B-27 that uses only defined humanized or recombinant components. It is worth noting that this cell culture system does not utilize animal-derived components and can therefore be adapted for any studies aiming to generate large numbers of differentiated SH-SY5Y cells without animal-derived reagents such as FBS, thereby increasing human relevance and translational ability. As the transition towards animal-free science is becoming more popular, having the option to gather preliminary data in human glutamatergic SH-SY5Y “neurons” could be a valuable tool for neuroscientists.

## Data Availability

The original contributions presented in the study are included in the article/Supplementary Material, further inquiries can be directed to the corresponding author.
