# Supplementary material for: A novel method for generating glutamatergic SH-SY5Y neuron-like cells utilizing B-27 supplement
Source: Front Pharmacol. 2022 Oct 20;13:943627. doi: 10.3389/fphar.2022.943627 (PMC9630362; doi:10.3389/fphar.2022.943627)
Supplement: Supplementary file 1 [file DataSheet1.docx]

Supplementary File 1


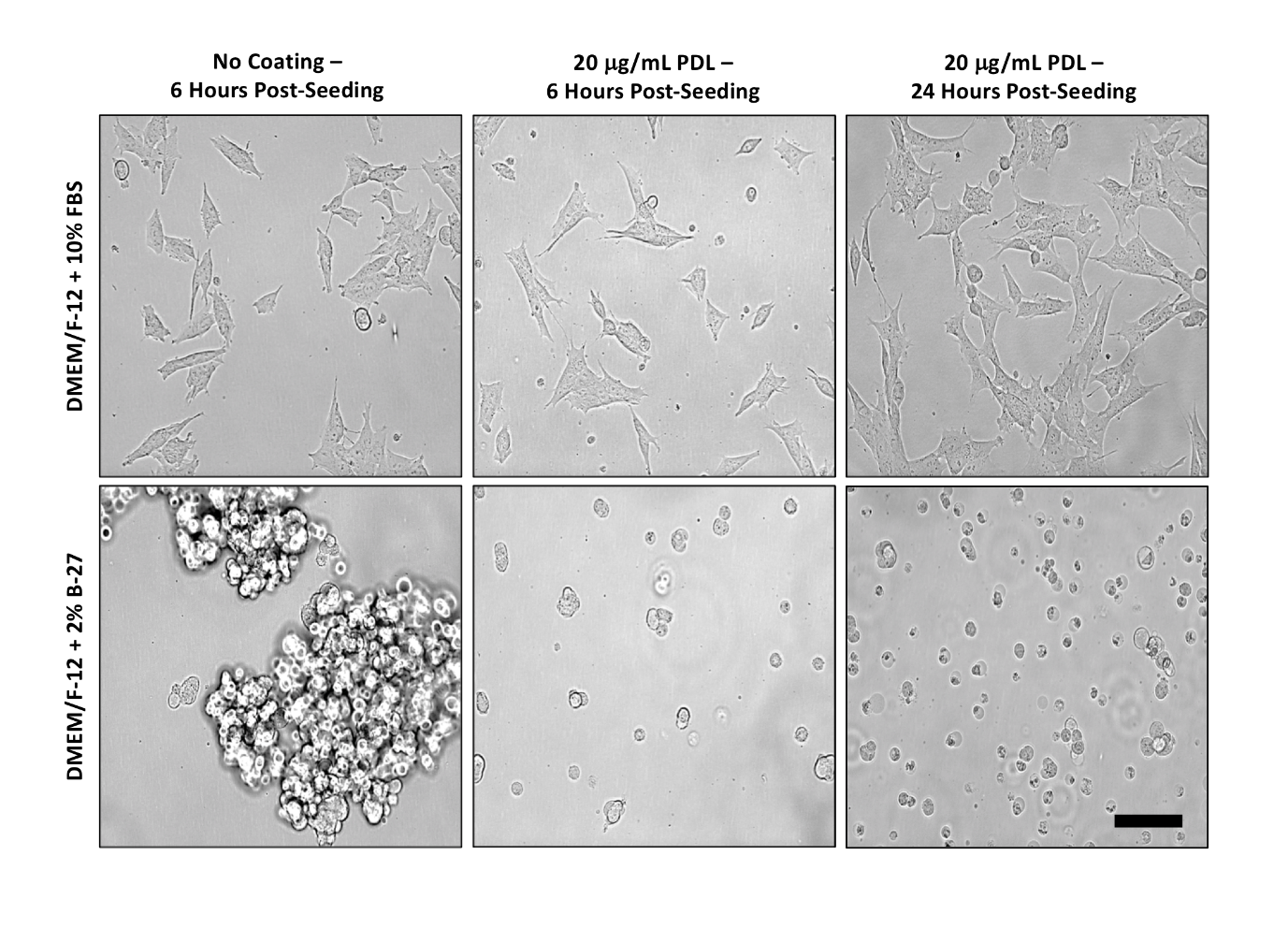


**Supplementary Figure 1: SH-SY5Y cells cultured in B-27 show attachment deficits following passaging, which are not rescued by pre-coating the surface with PDL.** SH-SY5Y cells were cultured in 6-well plates with either DMEM/F-12 + 10% FBS or DMEM/F-12 + 2% B-27 until they reached approximately 70% confluency. SH-SY5Y were then passaged in a 1:5 ratio into uncoated 6-well plates or 6-well plates pre-coated with 20 μg/mL PDL overnight at 37°C, with their respective media. Representative brightfield images were taken 6 hours and 24 hours after the cells were passaged and seeded into new 6-well uncoated/coated plates using the EVOS FLoid microscope (*Thermo Scientific*). Scale bar = 100 μm. DMEM/F-12: Dulbecco’s modified eagle medium/nutrient mixture F-12 with GlutaMAX supplement; FBS: Fetal bovine serum; PDL: Poly-D-lysine.
